# Integrated Analysis of Key Pathways and Drug Targets Associated With Vogt-Koyanagi-Harada Disease

**DOI:** 10.3389/fimmu.2020.587443

**Published:** 2020-12-15

**Authors:** Zhijun Chen, Zhenyu Zhong, Wanyun Zhang, Guannan Su, Peizeng Yang

**Affiliations:** The First Affiliated Hospital of Chongqing Medical University, Chongqing Key Laboratory of Ophthalmology, and Chongqing Eye Institute, Chongqing, China

**Keywords:** Vogt-Koyanagi-Harada disease, pathway analysis, network analysis, drug repurposing, uveitis

## Abstract

**Background:**

Vogt-Koyanagi-Harada (VKH) disease is a complex disease associated with multiple molecular immunological mechanisms. As the underlying mechanism for VKH disease is unclear, we hope to utilize an integrated analysis of key pathways and drug targets to develop novel therapeutic strategies.

**Methods:**

Candidate genes and proteins involved in VKH disease were identified through text-mining in the PubMed database. The GO and KEGG pathway analyses were used to examine the biological functions of the involved pathways associated with this disease. Molecule-related drugs were predicted through Drug-Gene Interaction Database (DGIdb) analysis.

**Results:**

A total of 48 genes and 54 proteins were associated with VKH disease. Forty-two significantly altered pathways were identified through pathway analysis and were mainly related to immune and inflammatory responses. The top five of significantly altered pathways were termed as “inflammatory bowel disease,” “cytokine-cytokine receptor interaction,” “allograft rejection,” “antigen processing,” and “presentation and Herpes simplex infection” in the KEGG database. IFN-γ and IL-6 were identified as the key genes through network analysis. The DGIdb analysis predicted 48 medicines as possible drugs for VKH disease, among which Interferon Alfa-2B was co-associated both with IFN-γ and IL-6.

**Conclusions:**

In this study, systematic analyses were utilized to detect key pathways and drug targets in VKH disease *via* bioinformatics analysis. IFN-γ and IL-6 were identified as the key mediators and possible drug targets in VKH disease. Interferon Alfa-2B was predicted to be a potentially effective drug for VKH disease treatment by targeting IFN-γ and IL-6, which warrants further experimental and clinical investigations.

## Introduction

Vogt-Koyanagi-Harada (VKH) disease is an immune-mediated disorder characterized by chronic, bilateral granulomatous panuveitis, often associated with neurological, audiovestibular and cutaneous manifestations (1). VKH disease is more commonly seen in Asians, Hispanics, Native Americans (2), and rare in Africans (3). Bilateral panuveitis, hearing disorder and meningitis are the main clinical features. Treatment with systemic corticosteroid is the mainstay of VKH disease therapy in the acute uveitic phase (4). However, despite proper treatment with a high-dose of corticosteroid, 79% of patients will experience recurrent attacks and develop chronic disease (5). Moreover, a high-dose of corticosteroid over a prolonged period may lead to side effects such as Cushing syndrome, hyperglycemia, and increased incidence of severe infections (6). Currently, there remain unmet medical needs for novel therapies that can etiologically target the molecules or immune mediators involved in the disease.

Although the exact biological mechanisms are still unclear, an increasing number of candidate genes and proteins have been reported to be involved in the development of VKH disease, which may be possible drug targets for the disease (7, 8). However, it is still challenging to prioritize these drug targets among many genes and proteins. Here, we used integrated bioinformatic analysis to summarize the candidate genes and proteins associated with VKH disease and identify the potential key pathways and drug targets, which may help to develop new therapeutic agents.

## Materials and Methods

### Identifying Candidate Genes and Proteins Associated With VKH Disease

We manually collected candidate genes and proteins associated with VKH disease by a thorough review of the literature in any language published from May 1981 to November 2019, using a similar approach used earlier by others (9, 10). We used the following terms to search the PubMed database: “idiopathic uveoencephalitis” OR uveoencephalitis OR “uveomeningitic syndrome” OR Vogt-Koyanagi-Harada. Studies were eligible if they included a comparison of the expression levels of a gene or protein between VKH patients and controls. Key exclusion criteria included: (i) studies only conducted in animal models, (ii) meta-analysis of published results, and (iii) review and comment papers. We also matched eligible genes and proteins with our previously published database (UVEOGENE, http://www.uvogene.com) (11). Two researchers were trained in each step with pilot tests before collecting and managing data independently. Regular meetings were held to clear up any misunderstandings or disagreements.

### Functional Analysis

Functional analysis was performed based on the DAVID online tools (version 6.8, http://david.ncifcrf.gov). In the gene ontology (GO) database, the analysis of candidate genes and proteins was divided into three categories, termed as biological processes (BP), molecular functions (MF), and cellular components (CC) (12). The GO database provides annotations to describe the properties of genes and gene products of different organisms and shows enriched genes’ potential function. BP is an ordered combination of molecular functions to describe a wide range of biological processes. The MF is used to describe the function of a gene or gene product, and the CC is designed for describing subcellular structures, locations, and macromolecular complexes of genes. We submitted the list of identified candidate genes to the DAVID online tools and obtained the significant enrichment of the above categories. A significant threshold of *P* < 0.05 was used.

### KEGG Pathway Enrichment Analysis

Kyoto Encyclopedia of Genes and Genomes (KEGG) pathway enrichment analysis was performed on candidate genes to enrich significantly altered pathways, using the DAVID online tools regarding the KEGG database. KEGG is an encyclopedia of genes and genomes for understanding high-level biological functions and utilities (13). The KEGG database provides a collection of graphical diagrams (pathway maps), in which some of the known metabolic pathways and regulatory pathways are displayed to describe the linkage, interaction and function of enriched candidate genes across different cells and organisms. In this study, enriched pathways were considered significant if including at least two genes and reaching a significance level of *P* < 0.05.

### Protein-Protein Interaction Analysis

Protein-protein interaction (PPI) analysis was performed based on candidate genes and proteins using the STRING database (https://stringdb.org/). The STRING database aims to collect and integrate the information by integrating known and predicted protein-protein association data from many organisms (14). Default parameters in STRING were used. Cytoscape software (version 3.7.2, California, USA) was used to construct and visualize the PPI network. Cytoscape is a graphical display tool used for visualizing complex biological networks (15). Furthermore, we used the Cytoscape plugin molecular complex detection (MCODE) to explore significant modules in the PPI network using the following scores and parameters: k score= 2, degree cutoff= 2, node score cutoff= 0.2, and maximum depth= 100 (16).

### Identification of Hub Genes

CytoHubba plugin in Cytoscape was used to identify hub genes in the PPI network by calculating and analyzing the network structure. Three algorithms were used to generate intersecting genes, including the maximum neighborhood component (MNC), the degree, and the closeness, as described previously (17). Briefly, the degree is the number of the edges of a gene in the network, representing the interaction pairs with others. The closeness could be used to evaluate the genes in the network according to the calculated centrality. The MNC represents the number of nodes in the maximum connected subgraph. The genes generated by the above algorithms’ intersection were more likely to locate in a core position and were considered as the hub genes.

### Drug-Gene Interactions

The Drug-Gene Interaction Database (DGIdb, http://dgidb.genome.wustl.edu/) is a drug prediction database, which can be used to screen drugs that potentially target certain genes of interest (18). The DGIdb provides gene-drug interactions and potential druggability according to their gene category. We utilized the DGIdb analysis to obtain all possible gene-drug interactions for the top-two ranked molecules of hub genes. Cytoscape was used to visualize the acquired interactions.

## Results

### Genes and Proteins Acquisition

**Figure 1:**
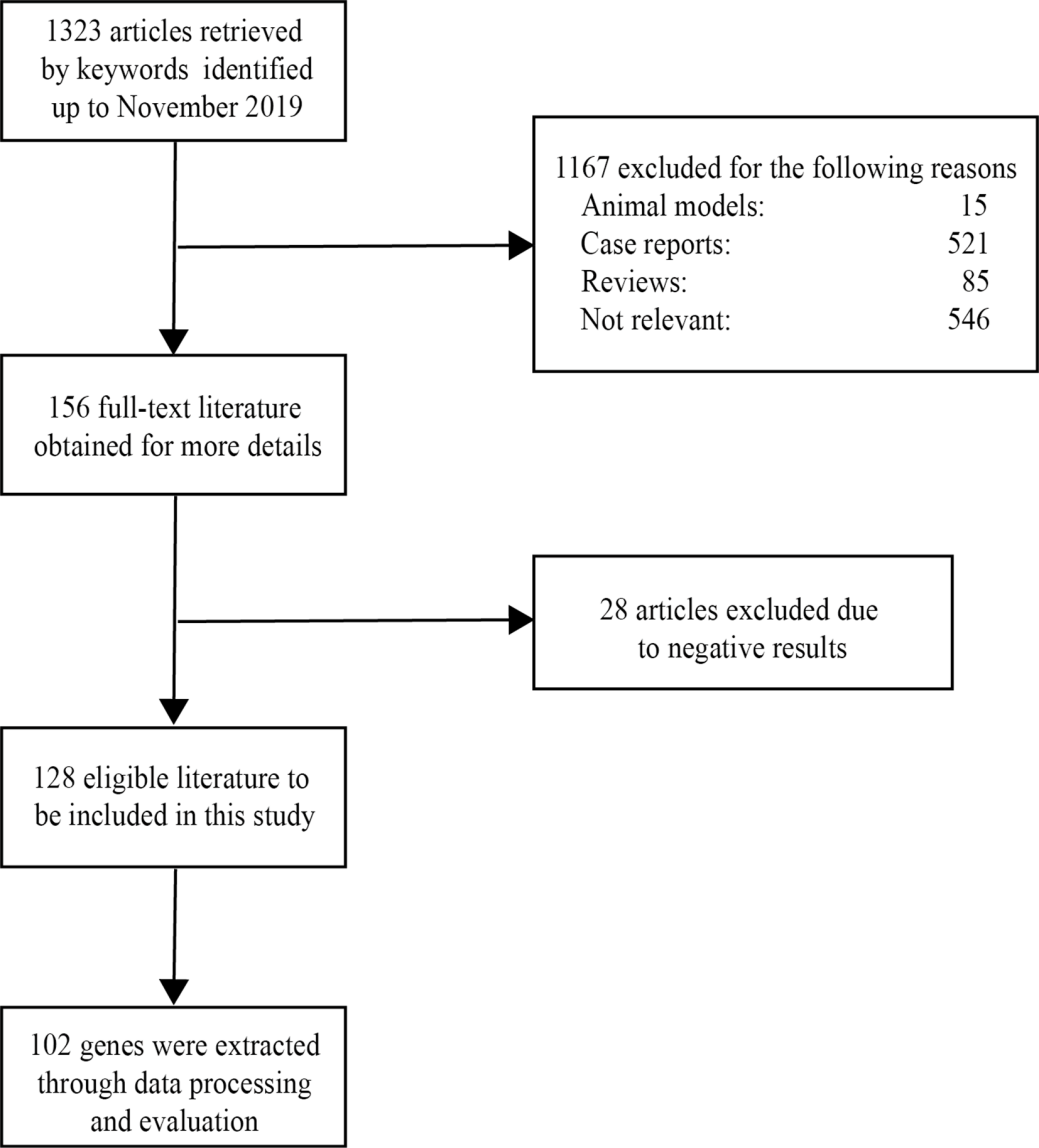
The flowchart of this study.

After screening a total of 1,323 articles from PubMed, we obtained 128 eligible articles. Based on the inclusion and exclusion criteria, we identified 48 genes and 54 proteins associated with VKH disease (**Table 1**). The expression of these candidate genes was reported to be significantly changed in VKH disease as compared with healthy individuals. Besides, among these candidate genes, 36 genes overlapped with genes recorded in the Uveogene database for VKH disease (11). These 36 genes had been reported to have susceptible single nucleotide polymorphisms and loci for VKH disease in the Uveogene database. **Figure 1** illustrates the flow chart of this study.

**Table 1:** The genes selected from eligible articles associated with VKH disease.

Number	Gene Symbol	Description	Gene ID of NCBI	Described in the Uveogene database yes or not
1	DRB1	RNA-binding protein 45	129831	Not
2	C3	complement C3	718	Yes
3	FCRL3	Fc receptor like 3	115352	Yes
4	PDCD1/PD1	programmed cell death 1	5133	Yes
5	IL23R	interleukin 23 receptor	149233	Yes
6	HLA-DRA	major histocompatibility complex, class II, DR alpha	3122	Yes
7	HLA-DRB5	major histocompatibility complex, class II, DR beta 5	3127	Not
8	ADO	2-aminoethanethiol dioxygenase	84890	Yes
9	ZNF365	zinc finger protein 365	22891	Not
10	EGR2	early growth response 2	1959	Not
11	SUMO4	small ubiquitin like modifier 4	387082	Yes
12	CYP2R1	cytochrome P450 family 2 subfamily R member 1	120227	Not
13	KIR 3DS1	killer cell immunoglobulin like receptor, three Ig domains and short cytoplasmic tail 1	3813	Not
14	KIR 2DS1	killer cell immunoglobulin like receptor, two Ig domains and short cytoplasmic tail 1	3806	Not
15	KIR 2DS5	killer cell immunoglobulin like receptor, two Ig domains and short cytoplasmic tail 5	3810	Not
16	KIR 3DL1	killer cell immunoglobulin like receptor, three Ig domains and long cytoplasmic tail 1	3811	Not
17	KIR B	killer- cell immunoglobulin- like receptor B	3805	Not
18	aKIR	akirin 2	55122	Not
19	JAK1	Janus kinase 1	3716	Yes
20	JAK2	Janus kinase 2	3717	Yes
21	STAT3	signal transducer and activator of transcription 3	6774	Yes
22	MIR146A	microRNA 146a	406938	Yes
23	ETS1	ETS proto-oncogene 1, transcription factor	2113	Yes
24	CFH	complement factor H	3075	Yes
25	KIAA1109	KIAA1109	84162	Yes
26	IL27	interleukin 27	246778	Yes
27	TGFBR3	transforming growth factor beta receptor 3	7049	Yes
28	CD40	CD40 molecule	958	Yes
29	TLR9	toll like receptor 9	54106	Yes
30	NLRP1	NLR family pyrin domain containing 1	22861	Yes
31	CLEC16A	C-type lectin domain containing 16A	23274	Yes
32	PTPN22	protein tyrosine phosphatase non-receptor type 22	26191	Yes
33	IFN-γ/IFN Gamma	interferon gamma	3458	Yes
34	BACH2	BTB domain and CNC homolog 2	60468	Yes
35	C1orf141	chromosome 1 open reading frame 141	400757	Not
36	CTLA4	cytotoxic T-lymphocyte associated protein 4	1493	Yes
37	UBLCP1	ubiquitin like domain containing CTD phosphatase 1	134510	Yes
38	IL12B	interleukin 12B	3593	Not
39	C2	complement C2	717	Not
40	CFB	complement factor B	629	Yes
41	CFI	complement factor I	3426	Yes
42	IL17F	interleukin 17F	112744	Yes
43	IL12RB2	interleukin 12 receptor subunit beta 2	3595	Not
44	MCP-1/CCL2	C-C motif chemokine ligand 2	6347	Yes
45	CCR6	C-C motif chemokine receptor 6	1235	Yes
46	FGFR1OP	centrosomal protein 43	11116	Yes
47	TNFAIP3	TNF alpha induced protein 3	7128	Yes
48	TRAF5	TNF receptor associated factor 5	7188	Yes
49	IL25	Interleukin-25	64806	Not
50	HLA-DRB4	HLA class II histocompatibility antigen, DR beta 4 chain	3126	Not
51	IGHD	Immunoglobulin heavy constant delta	3495	Not
52	TGFBR2	TGF-beta receptor type-2	7048	Not
53	PSIP1	PC4 and SFRS1-interacting protein	11168	Not
54	HLA-B	HLA class I histocompatibility antigen B alpha chain	3106	Not
55	UACA	Uveal autoantigen with coiled-coil domains and ankyrin repeats	55075	Not
56	PAPSS2	Bifunctional 3'-phosphoadenosine 5'-phosphosulfate synthase 2	9060	Not
57	CXCL10	C-X-C motif chemokine 10	3627	Yes
58	CD4	T-cell surface glycoprotein CD4	920	Not
59	HLA-DPB1	HLA class II histocompatibility antigen, DP beta 1 chain	3115	Not
60	TNFSF13	Tumor necrosis factor ligand superfamily member 13	8741	Not
61	CD3E	T-cell surface glycoprotein CD3 epsilon chain	916	Not
62	GPBAR1	G-protein coupled bile acid receptor 1	151306	Not
63	HLA-DRB1	major histocompatibility complex, class II, DR beta 1	3123	Yes
64	BCL2A1	Bcl-2-related protein A1	597	Not
65	CXCL9	C-X-C motif chemokine 9	4283	Not
66	LEP	Leptin	3952	Not
67	AGER	Advanced glycosylation end product-specific receptor	177	Not
68	FAS	Tumor necrosis factor receptor superfamily member 6	355	Not
69	IL35	Interleukin-35	3592	Not
70	TYR	Tyrosinase	7299	Not
71	IL23A	Interleukin-23 subunit alpha	51561	Not
72	HLA-DQA1	HLA class II histocompatibility antigen, DQ alpha 1 chain	3117	Not
73	ARMC9	armadillo repeat containing 9	80210	Not
74	IL9	Interleukin-9	3578	Not
75	C4B	Complement C4-B	721	Not
76	IL21	Interleukin-21	59067	Not
77	IL6	Interleukin-6	3569	Not
78	C3AR1	C3a anaphylatoxin chemotactic receptor	719	Not
79	IL2RA	Interleukin-2 receptor subunit alpha	3559	Not
80	CCL8	C-C motif chemokine 8	6355	Not
81	DAB2	Disabled homolog 2	1601	Not
82	KIR2DS3	Killer cell immunoglobulin-like receptor 2DS3	3808	Not
83	SPP1	Osteopontin	6696	Yes
84	NOD1	Nucleotide-binding oligomerization domain-containing protein 1	10392	Not
85	IL37	Interleukin-37	27178	Not
86	HLA-DQB1	HLA class II histocompatibility antigen, DQ beta 1 chain	3119	Not
87	FOXP3	Forkhead box protein P3	50943	Not
88	IL7	Interleukin-7	3574	Not
89	IRAK1	Interleukin-1 receptor-associated kinase 1	3654	Not
90	HLA-A	MHC class I antigen	3105	Not
91	IL4	Interleukin-4	3565	Not
92	MIF	Macrophage migration inhibitory factor	4282	Not
93	TLR3	Toll-like receptor 3	7098	Not
94	KIR2DS2	Killer cell immunoglobulin-like receptor 2DS2	100132285	Not
95	VEGFA	Vascular endothelial growth factor A	7422	Not
96	ESD	S-formylglutathione hydrolase	2098	Not
97	CXCL1	C-X-C motif chemokine ligand 1	2919	Not
98	FCGBP	Fc fragment of IgG binding protein	8857	Not
99	PAX3	Paired box 3	5077	Not
100	CXCL13	C-X-C motif chemokine 13	10563	Not
101	IL15	Interleukin-15	3600	Not
102	IL1B	Interleukin-1 beta	3553	Not

### GO Analysis of Genes and Proteins

**Figure 2:**
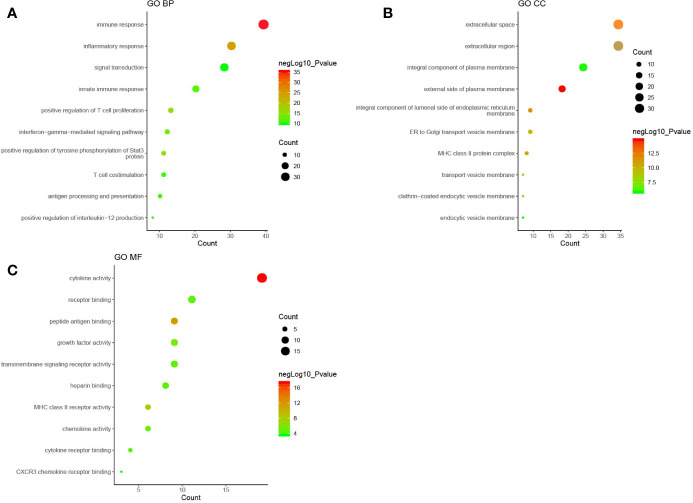
The top ten functional enrichment analyses of gene ontology (GO). **(A)**: biological processes (BP); **(B)**: cellular components (CC); **(C)**: molecular functions (MF). Each circle in **Figure 2** represented a pathway of GO analysis. The pathway highlighted in red represented more significant P-values, whereas the pathway highlighted in green represented less significant P-values. The size of circle represented the count of pathway, and a larger circle indicated a larger count enriched in the pathway.

GO enrichment analysis was performed with the DAVID online tools to examine the identified genes and proteins’ biological characteristics. The analysis of BP (biological processes) showed a total of 251 functions, 200 of which were significantly enriched (*P* < 0.05). The top-ranked functions included the categories “immune response,” “inflammatory response,” “positive regulation of T cell proliferation,” “positive regulation of tyrosine phosphorylation of Stat3 protein,” and “interferon-gamma-mediated signaling pathway.” The CC (cellular components) analysis included 29 functions, of which 26 were significantly enriched (*P* < 0.05). For the CC analysis, the identified genes and proteins were mostly enriched in the “external side of plasma membrane,” “extracellular space,” “integral component of luminal side of endoplasmic reticulum membrane,” “extracellular region,” and “MHC class II protein complex.” The MF (molecular functions) analysis included 37 functions, 26 of which were significantly enriched (*P* < 0.05). Changes in MF were significantly enriched in “cytokine activity,” “peptide antigen binding,” “MHC class II receptor activity,” “growth factor activity,” and “chemokine activity.” The top ten functional enrichment analyses of GO (BP, MF, CC) are shown in **Figure 2**, and the significant GO (BP, MF, CC) are provided in **Supplementary Table S1**. The corresponding genes enriched in GO analysis (**Figure 2**) are listed in **Supplementary Table S2**.

### KEGG Analysis of Genes and Proteins

**Figure 3:**
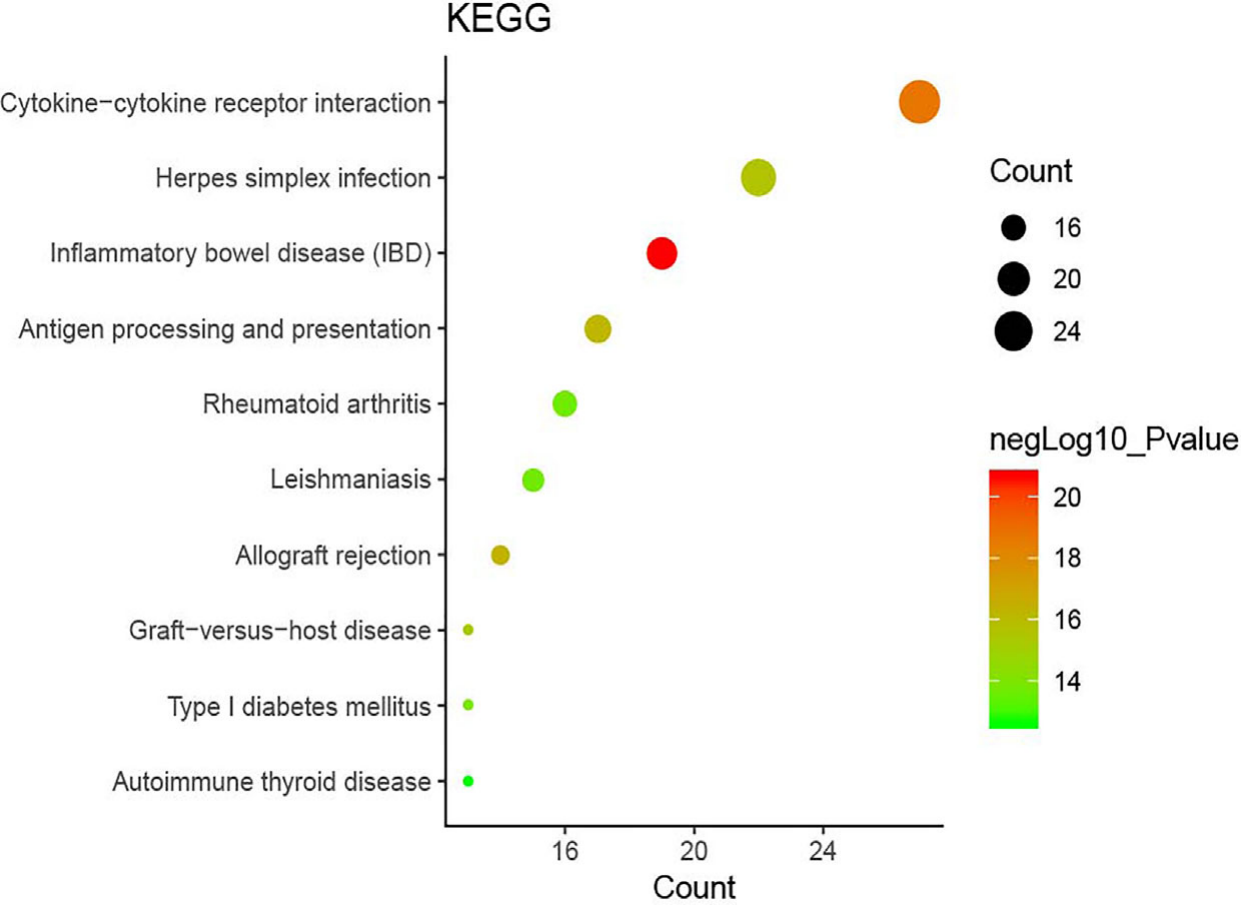
The top ten pathway enrichment analyses from the Kyoto Encyclopedia of Genes and Genomes (KEGG) database.

**Figure 4:**
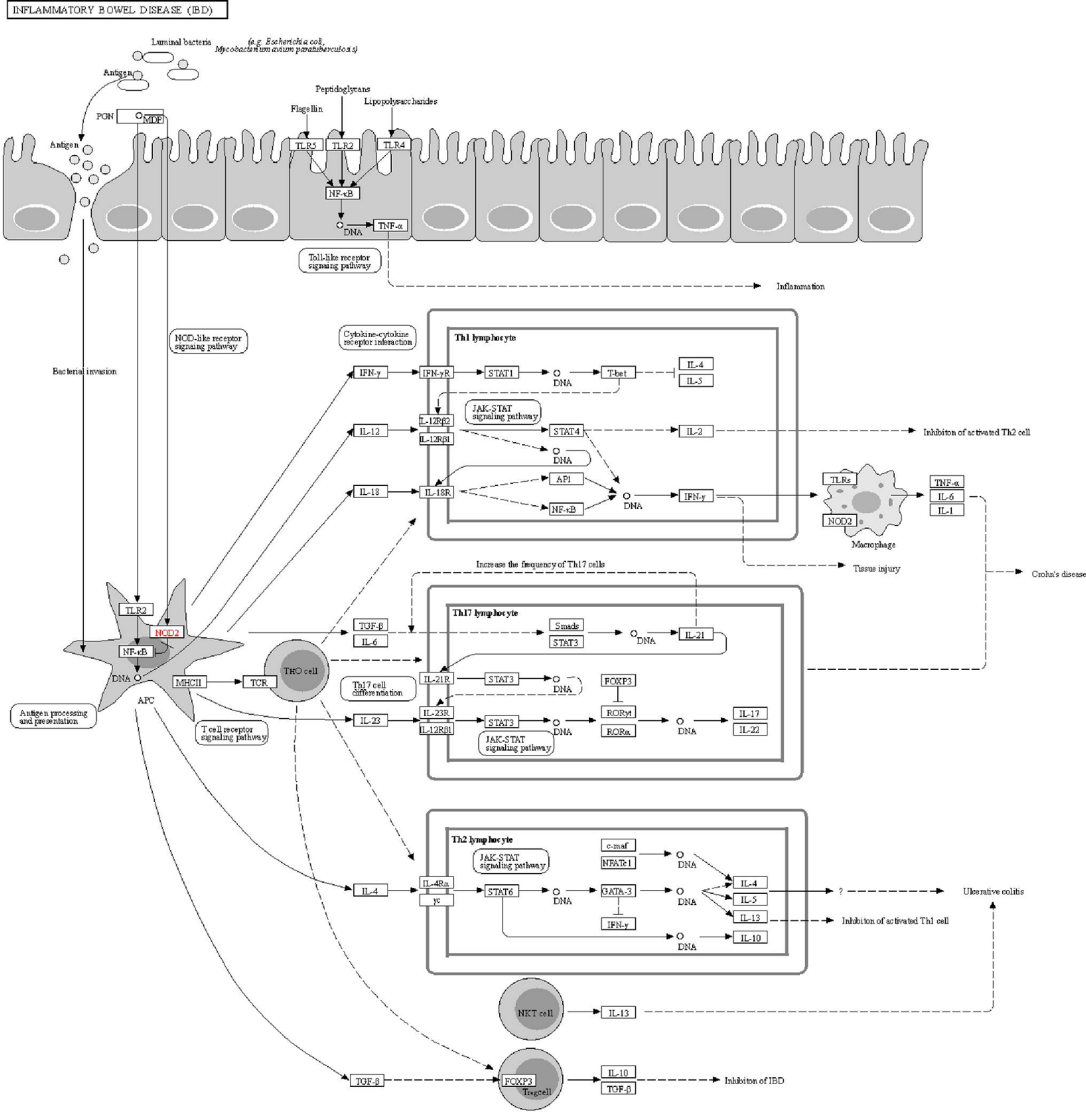
The KEGG pathway schematic diagram of inflammatory bowel disease (IBD).

**Figure 5:**
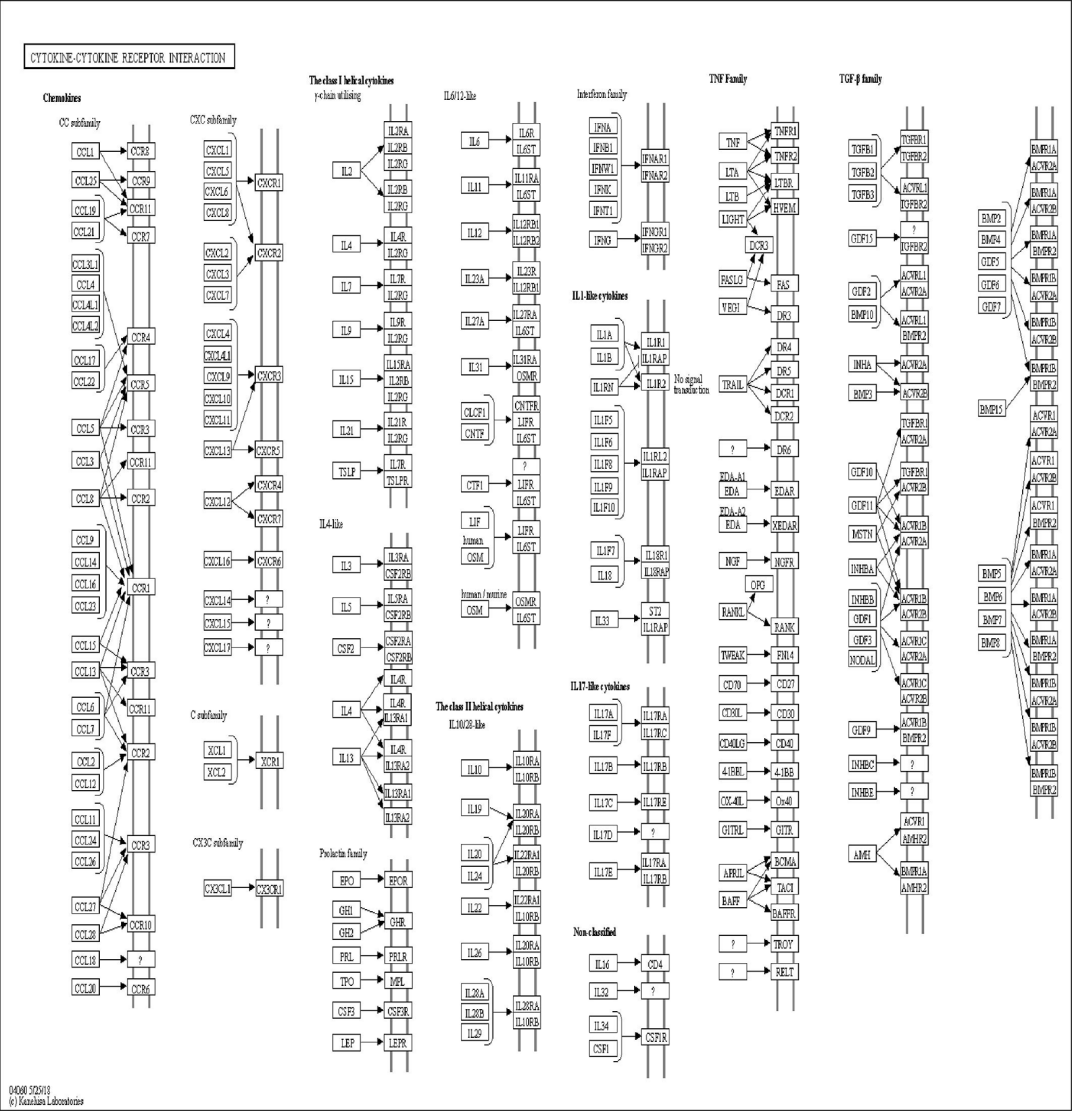
The KEGG pathway schematic diagram of cytokine-cytokine receptor interaction.

The DAVID online tools were utilized for the KEGG enrichment pathway analysis to show the potential involvement of pathways related to identified candidate genes and proteins. The analysis of KEGG identified 42 significantly altered pathways (*P* < 0.05) (**Supplementary Table S3**). The top-ranked pathways were mainly involved in categories termed as “inflammatory bowel disease (IBD),” “cytokine-cytokine receptor interaction,” “allograft rejection,” “antigen processing,” and “presentation and Herpes simplex infection” (**Figure 3**). The corresponding genes enriched in the KEGG pathway (**Figure 3**) are listed in **Supplementary Table S4**. Additionally, the IBD pathway was the most significant in the enrichment analysis with 19 genes involved (**Figure 4**). The cytokine-cytokine receptor interaction had the largest number of genes, 27 genes enriched in the pathway (**Figure 5**). Moreover, several genes, including IFN-γ, IL6, IL12, IL4, IL23R and IL21, were shared by the IBD pathway and the cytokine-cytokine receptor interaction pathway and were related to the signaling transductions by members of the interleukin-family indicating that these members of the interleukin-family might play an important role in the pathogenesis of VKH disease.

### PPI Network Analysis and Related Gene Modules

**Figure 6:**
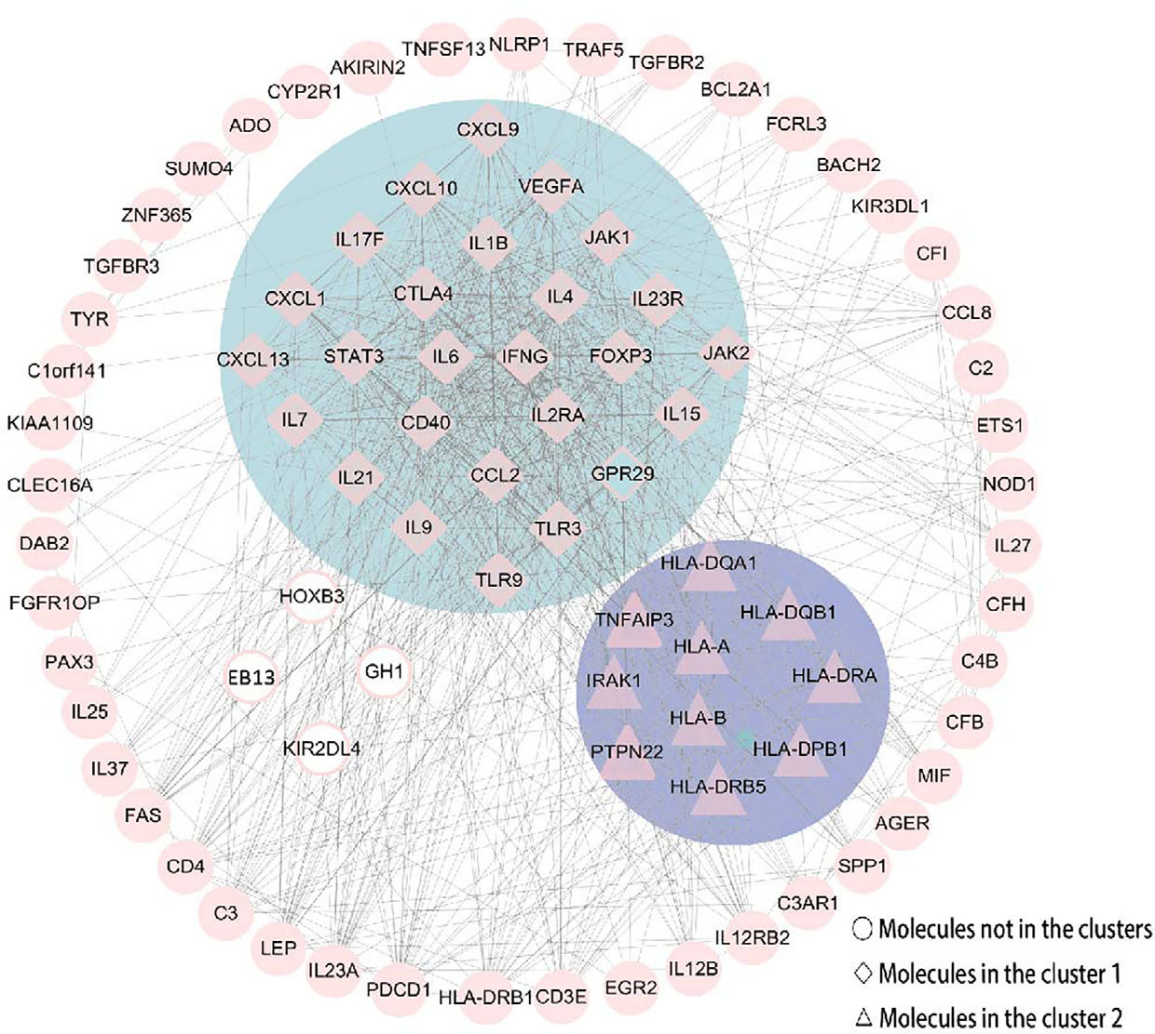
The PPI network was constructed by STRING and Cytoscape. There were 87 nodes and 754 edges in the PPI network. Besides, two modules were recognized by MCODE in Cytoscape. Cluster 1 (score = 23.28) was the most powerful module containing 26 nodes and 291 edges. Cluster 2 (score = 6.889) consisted of 10 nodes and 31 edges. Molecules represented as a pink diamond in the circle with a blue background represents genes belonging to cluster 1, pink triangles in the circle with a purple background represent genes belonging to cluster 2, and genes in the white background do not belong to either cluster. HOXB3, KIR2DL4, GH1, EBI3 and GPR29 in pink graphics were predicted genes using interactions between submitted genes in the PPI network by Cytoscape, and other nodes were of submitted genes in the PPI network and showed interactions between multiple genes.

The PPI network consisted of STRING and Cytoscape, which were used to determine the most important genes and proteins clusters. All the 87 nodes and 754 edges in the PPI network are shown in **Figure 6**. Besides, the MCODE plugin in Cytoscape identified two main modules. Cluster 1 (score = 23.28) consisted of 26 nodes and 291 edges, and cluster 2 (score = 6.889) consisted of 10 nodes and 31 edges (**Figure 6**). In cluster 1, several genes, including IFN-γ, IL6, IL4, IL23R and IL21, were significantly enriched. These genes were also related to the enriched pathways, including the IBD pathway and the cytokine-cytokine receptor interaction pathway. In cluster 2, the most enriched genes were related to the human leukocyte antigen (HLA) family, including HLA-A, HLA-B, HLA-DQA1, HLA-DQB1, HLA-DRA, HLA-DPB1 and HLA-DRB5.

### Hub Genes Recognition

**Figure 7:**
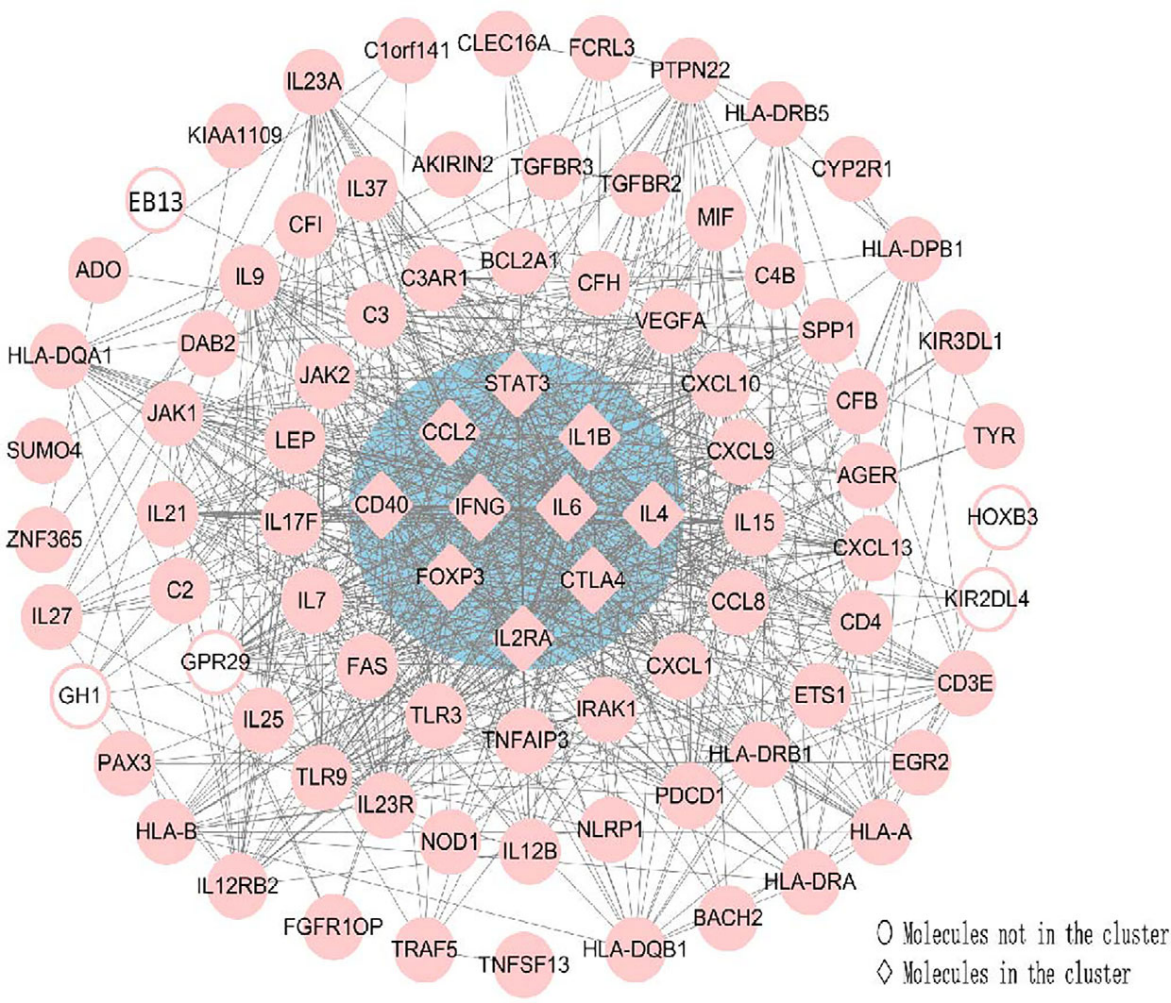
Using Cytohubba, a plugin Cytoscape, the top 10 genes were identified as hub genes. This analysis revealed two key genes: IFN-γ and IL-6 in the cluster. All the gene nodes and edges were calculated. Molecules represented as pink diamonds in the circle with a blue background represented genes belonging to the cluster showing the top ten genes in the network by the Cytoscape plugin Cytohubba, and pink circles without a colored background represent genes not belonging to this cluster. HOXB3, KIR2DL4, GH1, EBI3 and GPR29 in pink graphics were predicted genes using interactions between submitted genes in the PPI network by Cytoscape, and other nodes were of submitted genes in the PPI network and showed interactions between multiple genes.

CytoHubba was utilized to search for the key genes and calculated all the molecular nodes and edges. Consequently, 102 target genes were involved in the PPI network complex, forming 87 nodes and 754 edges (**Figure 7**). To search for the important nodes in the PPI network, all nodes were ranked by the three algorithms, including the degree, closeness and MNC provided by cytoHubba. The cytoHubba plugin Cytoscape was used to analyze the hub genes in the PPI network, and the following genes with the top ten grades were identified as hub genes: IL6, IFN-γ, IL4, CTLA4, IL1B, STAT3, CCL2, CD40, FOXP3 and IL2RA. Among these, IFN-γ and IL-6 were the top two of the ten grades and considered to be the key genes in this model, given the fact that the products of genes were at the core of the PPI network (**Figure 7**). The descriptions of gene symbols and the details shown in **Figure 7** are provided in **Supplementary Table S5**.

### Drug-Gene Interactions

**Figure 8:**
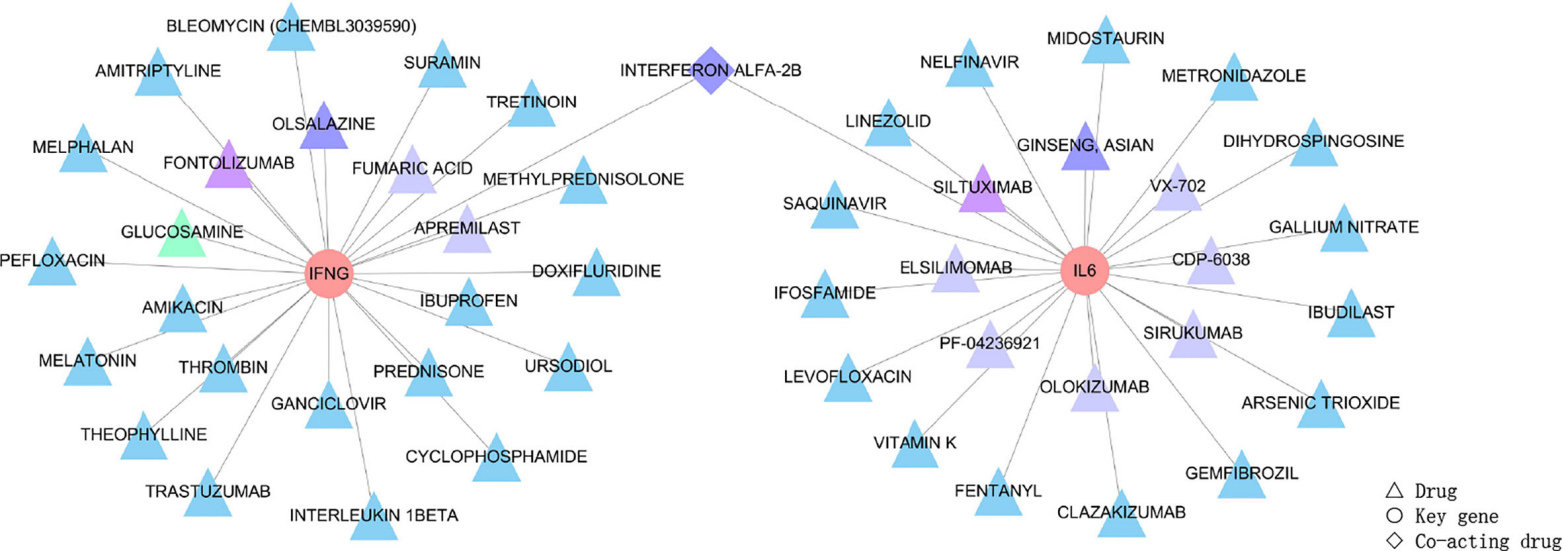
Drug targeting the two genes, IFN-γ and IL-6, shown as pink circles. 48 drugs as the main therapeutic agents shown in triangles and expressed in different groups according to scores. Firstly, the triangle in green represented GLUCOSAMINE obtained five scores through the DGIdb analysis, and it was the most relevant drug. Secondly, triangles in purple, including FONTOLIZUMAB and SILTUXIMAB acquired four scores, and these were the second most relevant drugs. Thirdly, drugs with three scores were OLSALAZINE and GINSENG ASIAN as shown by dark blue triangles, and INTERFERON ALFA-2B shown as dark blue diamonds since Interferon Alfa-2B was able to target both of the two key genes. Moreover, most drugs obtained two scores and are shown as light blue triangles (the target drugs with scores can be seen in **Supplementary Table S6**). Lastly, eight drugs, including FUMARIC ACID, APREMILAST, VX-702, CDP-6038, SIRUKUMAB, OLOKIZUMAB, ELSILIMOMAB and PF-04236921, obtained one score and are shown as blue-grey triangles. The scores of 48 drugs identified by the DGIdb show the importance of drugs, with a higher score indicating greater importance.

IFN-γ and IL-6, as the identified key genes, were entered into the DGIdb to obtain potential drugs. 48 drugs were identified in the DGIdb analysis; the information about the source, scores, and interaction type of target drugs is provided in the **Supplementary Table S6**. Most of the target drugs were inhibitors, monoclonal antibodies and immunomodulatory agents. Among these identified drugs, Interferon Alfa-2B was predicted to act on both IFN-γ and IL-6 (**Figure 8**).

## Discussion

In the present study, we used systematic analyses to show key pathways and potential VKH disease drugs. Two significant modules and a top ten hub genes were detected with IFN-γ and IL-6 as key genes. 48 target drugs were potentially useful drugs for the treatment of VKH disease. Interferon Alfa-2B targeted both IFN-γ and IL-6 and predicted a potentially useful drug to treat VKH, and warrants further experimental and clinical investigations.

The results of GO enrichment analysis indicated that immune response and inflammatory response play a significant role in VKH disease, which confirms earlier studies in this field (19–23). There still are many mediators related to these pathways, which have not been reported to be associated with VKH disease. Our enrichment analysis suggests that these molecules along with their involved pathways are closely linked with the development and pathogenic processes of VKH disease, which warrants further experimental investigations. Various mediators, including HOXB3, GH1, KIR2DL4 have not been reported in VKH disease, but these were predicted to have a high relevance for VKH disease as shown by our PPI network analysis. Previous studies have suggested that HOXB3, GH1 and KIR2DL4 are involved in autoimmune disease such as Thyroid-associated orbitopathy (TAO), Type 1 diabetes (T1DM), and Systemic lupus erythematosus (SLE) (24–26). Their functional role in VKH disease requires further studies. We also identified a variety of pathways related to certain autoimmune diseases such as the IBD pathway, the Toll-Like receptor pathway, the CD27–CD70 pathway and the CD40–CD40L pathway which have been shown to be associated with systemic lupus erythematosus (SLE), multiple sclerosis (MS), rheumatoid arthritis (RA), systemic sclerosis (SSc), Sjögren’s syndrome (SS), psoriasis, uveitis and other autoimmune diseases (11, 27–29). These findings suggest a certain degree of shared pathogenic pathways between VKH disease and other autoimmune diseases.

Two key genes, IFN-γ and IL-6, were identified in our study by cytoHubba, a plug-in Cytoscape. Lymphocytes produce IFN-γ in response to various immune stimuli. The essential role of IFN-γ in human anti-viral immunity has been illustrated earlier (30, 31). Several studies have indicated that VKH is an autoimmune disease mediated by Th1/IFN-γ and Th17/IL-17 pathways (32). Examples include studies that showed that the expression level of IFN-γ is significantly higher in peripheral blood mononuclear cell (PBMC), aqueous or serum of VKH patients as compared with those in control subjects (33–36). Besides IFN-γ we also identified the cytokine IL-6 as a key player in VKH disease. IL-6 is a four-helix cytokine composed of 184 amino acids (37) with various physiological functions, including regulating the proliferation and differentiation of immune cells. IL-6 modulates almost all aspects of the innate immune system. It has been shown that IL-6 plays a significant role in regulating the balance between IL-17 producing Th17 cells and regulatory T cells (Treg) (38, 39). Both T-cell subsets play an important role in the pathogenesis of VKH disease. Several studies have shown that the concentration of IL-6 in PBMC, monocyte-derived macrophages (MDMs), or aqueous humor from VKH patients is significantly higher than that observed in controls (40–42). This evidence support that IFN-γ and IL-6 are key mediators related to VKH disease, which suggests that they might be an attractive drug target for this disease. According to the analysis of the DGIdb, Interferon Alfa-2B is specific for both IFN-gamma and IL-6. The Drug-Gene Interaction database (DGIdb) mines available resources and predicts potentially effective therapeutic targets or prioritized drug development based on specific genes (18, 43, 44). We performed drug-gene interaction networks through bioinformatics analysis to identify target drugs that may act on both IFN-γ and IL-6. Of the 48 target drugs obtained from the DGIdb, most were inhibitors, monoclonal antibodies or immunomodulators. Among these potential drugs, Interferon Alfa-2B was found to be the drug that could target both IFN-γ as well as IL-6. The DGIdb provides evidence showing that Interferon Alfa-2B affects the expression of both IFN-γ and IL-6 (45, 46). Interferon, a class of cytokines, can interfere with virus replication, reduce cell proliferation, and alter immunity (47, 48). In recent studies, the role of IFN-Alfa in the pathogenesis of autoimmune diseases has been recognized (49, 50). Interferon Alfa-2B is an effective drug for treating autoimmune diseases such as idiopathic thrombocytopenic purpura (ITP) and uveitis (51, 52). The use of Interferon Alfa-2B has also been reported in the treatment of severe chronic uveitis in patients with Behçet’s disease (53) and uveitic cystoid macular edema (54). VKH disease, along with Behçet’s disease and uveitic cystoid macular edema, are anatomically classified as non-infectious posterior or pan-uveitis and can be treated with the same class of immunomodulatory drugs such as cyclosporin A (55, 56). The efficacy of Interferon Alfa-2B in the treatment of VKH disease has not yet been reported, but our analyses highlight the potential of Interferon Alfa-2B for the treatment of this disease, which necessitates further studies.

This study has some major limitations. It should be noted that we were dependent on existing data by integrative bioinformatic analysis, and our analyses were based on currently available information obtained from existing research surveys, suggesting that new information from future studies may influence the results presented here. Due to the lack of available data, dynamic networks’ development is not yet based on a genetic-epigenetic association. Moreover, our analyses are largely exploratory, and these results need to be further confirmed by experimental *in vitro* and *in vivo* studies.

## Conclusion

In this study, systematic analyses were performed to identify key pathways and drug targets in VKH disease *via* bioinformatics analysis. Two significant modules and a top ten hub genes in VKH disease were detected with IFN-γ and IL-6 as the top two genes. The study furthermore predicted Interferon Alfa-2B as a potentially useful drug for the treatment of VKH disease.

## Data Availability Statement

The raw data supporting the conclusions of this article will be made available by the authors, without undue reservation.

## Author Contributions

PY and ZC conceived and designed the study. ZC and WZ did the literature review. ZZ and GS checked data. ZC and ZZ analyzed and interpreted the data. ZC wrote the first draft of the paper. PY supervised the study. All authors contributed to the article and approved the submitted version.

## Funding

This study was supported by National Natural Science Foundation Key Program (81930023), Natural Science Foundation Major International (Regional) Joint Research Project (81720108009), Chongqing Outstanding Scientists Project (2019), Chongqing Key Laboratory of Ophthalmology (CSTC, 2008CA5003), Chongqing Science & Technology Platform and Base Construction Program (cstc2014pt-sy10002) and the Chongqing Chief Medical Scientist Project (2018).

## Conflict of Interest

The authors declare that the research was conducted in the absence of any commercial or financial relationships that could be construed as a potential conflict of interest.
