# Supplementary material for: Integrated Analysis of Key Pathways and Drug Targets Associated With Vogt-Koyanagi-Harada Disease
Source: Front Immunol. 2020 Dec 15;11:587443. doi: 10.3389/fimmu.2020.587443 (PMC7769821; doi:10.3389/fimmu.2020.587443)
Supplement: Supplementary file 1 [file DataSheet_1.zip › Supplementary Table 1.DOCX]

**Supplementary Table S1** Significant functional analyses of gene ontology (GO) pathways.

| Number | GO classification | Pathway | P Value | Count |
| --- | --- | --- | --- | --- |
| 1 | BP | immune response | 3.57829E-36 | 39 |
| 2 | BP | inflammatory response | 3.24644E-25 | 30 |
| 3 | BP | positive regulation of T cell proliferation | 5.76618E-16 | 13 |
| 4 | BP | positive regulation of tyrosine phosphorylation of Stat3 protein | 8.56879E-15 | 11 |
| 5 | BP | interferon-gamma-mediated signaling pathway | 2.02963E-13 | 12 |
| 6 | BP | innate immune response | 2.91405E-12 | 20 |
| 7 | BP | T cell costimulation | 1.89611E-11 | 11 |
| 8 | BP | antigen processing and presentation | 2.06917E-11 | 10 |
| 9 | BP | positive regulation of interleukin-12 production | 6.55479E-11 | 8 |
| 10 | BP | signal transduction | 1.39033E-10 | 28 |
| 11 | BP | positive regulation of tumor necrosis factor production | 2.03954E-10 | 9 |
| 12 | BP | positive regulation of inflammatory response | 2.90696E-10 | 10 |
| 13 | BP | antigen processing and presentation of peptide or polysaccharide antigen via MHC class II | 3.28855E-10 | 7 |
| 14 | BP | T cell receptor signaling pathway | 7.10136E-10 | 12 |
| 15 | BP | response to lipopolysaccharide | 2.11925E-09 | 12 |
| 16 | BP | detection of bacterium | 6.47047E-09 | 6 |
| 17 | BP | positive regulation of interferon-gamma production | 6.62719E-09 | 8 |
| 18 | BP | regulation of complement activation | 1.48713E-08 | 7 |
| 19 | BP | positive regulation of tyrosine phosphorylation of Stat5 protein | 3.05587E-08 | 6 |
| 20 | BP | positive regulation of cell proliferation | 5.14083E-08 | 16 |
| 21 | BP | positive regulation of granulocyte macrophage colony-stimulating factor production | 1.96683E-07 | 5 |
| 22 | BP | positive regulation of activated T cell proliferation | 3.81239E-07 | 6 |
| 23 | BP | negative regulation of interleukin-10 production | 4.59601E-07 | 5 |
| 24 | BP | positive regulation of interferon-gamma biosynthetic process | 4.59601E-07 | 5 |
| 25 | BP | positive regulation of T cell mediated cytotoxicity | 6.60992E-07 | 5 |
| 26 | BP | positive regulation of interleukin-17 production | 6.60992E-07 | 5 |
| 27 | BP | regulation of immune response | 7.41557E-07 | 10 |
| 28 | BP | positive regulation of NF-kappaB transcription factor activity | 8.5442E-07 | 9 |
| 29 | BP | inflammatory response to antigenic stimulus | 1.25099E-06 | 5 |
| 30 | BP | immunoglobulin production involved in immunoglobulin mediated immune response | 1.73991E-06 | 4 |
| 31 | BP | cell chemotaxis | 1.76438E-06 | 7 |
| 32 | BP | negative regulation of T cell proliferation | 1.96852E-06 | 6 |
| 33 | BP | chemokine-mediated signaling pathway | 2.97703E-06 | 7 |
| 34 | BP | cell surface receptor signaling pathway | 3.38807E-06 | 11 |
| 35 | BP | positive regulation of activation of JAK2 kinase activity | 3.46553E-06 | 4 |
| 36 | BP | positive regulation of tissue remodeling | 3.46553E-06 | 4 |
| 37 | BP | defense response to protozoan | 3.49114E-06 | 5 |
| 38 | BP | positive regulation of interleukin-6 production | 5.32389E-06 | 6 |
| 39 | BP | positive regulation of NF-kappaB import into nucleus | 5.34417E-06 | 5 |
| 40 | BP | chemotaxis | 5.8513E-06 | 8 |
| 41 | BP | positive regulation of natural killer cell proliferation | 6.03978E-06 | 4 |
| 42 | BP | humoral immune response mediated by circulating immunoglobulin | 6.03978E-06 | 4 |
| 43 | BP | positive regulation of apoptotic cell clearance | 6.03978E-06 | 4 |
| 44 | BP | positive regulation of defense response to virus by host | 6.50351E-06 | 5 |
| 45 | BP | positive regulation of peptidyl-tyrosine phosphorylation | 6.9241E-06 | 7 |
| 46 | BP | positive regulation of transcription from RNA polymerase II promoter | 8.30064E-06 | 19 |
| 47 | BP | cytokine-mediated signaling pathway | 9.35477E-06 | 8 |
| 48 | BP | antigen processing and presentation of exogenous peptide antigen via MHC class II | 1.34747E-05 | 7 |
| 49 | BP | defense response to Gram-negative bacterium | 1.44974E-05 | 6 |
| 50 | BP | humoral immune response | 1.72945E-05 | 6 |
| 51 | BP | positive regulation of chemokine biosynthetic process | 2.04538E-05 | 4 |
| 52 | BP | response to antibiotic | 3.06151E-05 | 5 |
| 53 | BP | T-helper 1 type immune response | 3.71916E-05 | 4 |
| 54 | BP | defense response to virus | 4.16542E-05 | 8 |
| 55 | BP | cellular response to lipopolysaccharide | 4.34078E-05 | 7 |
| 56 | BP | negative regulation of apoptotic process | 5.15865E-05 | 12 |
| 57 | BP | positive regulation of B cell proliferation | 6.79362E-05 | 5 |
| 58 | BP | apoptotic process | 8.31357E-05 | 13 |
| 59 | BP | regulation of cell proliferation | 8.58704E-05 | 8 |
| 60 | BP | regulation of interleukin-10 secretion | 9.46645E-05 | 3 |
| 61 | BP | positive regulation of gene expression | 0.000118949 | 9 |
| 62 | BP | response to vitamin D | 0.000134589 | 4 |
| 63 | BP | positive regulation of osteoclast differentiation | 0.00015917 | 4 |
| 64 | BP | positive regulation of T-helper 17 cell lineage commitment | 0.00018863 | 3 |
| 65 | BP | regulation of interleukin-4 production | 0.00018863 | 3 |
| 66 | BP | regulation of tyrosine phosphorylation of Stat1 protein | 0.00018863 | 3 |
| 67 | BP | negative regulation of chronic inflammatory response | 0.00018863 | 3 |
| 68 | BP | positive regulation of tyrosine phosphorylation of Stat4 protein | 0.00018863 | 3 |
| 69 | BP | positive regulation of interleukin-10 production | 0.000286174 | 4 |
| 70 | BP | peptide antigen assembly with MHC class II protein complex | 0.000313224 | 3 |
| 71 | BP | positive regulation of NK T cell activation | 0.000313224 | 3 |
| 72 | BP | positive regulation of MAP kinase activity | 0.000344806 | 5 |
| 73 | BP | aging | 0.000347136 | 7 |
| 74 | BP | cellular defense response | 0.000417194 | 5 |
| 75 | BP | response to hypoxia | 0.00043319 | 7 |
| 76 | BP | positive regulation of memory T cell differentiation | 0.000468103 | 3 |
| 77 | BP | positive regulation of T-helper 17 type immune response | 0.000468103 | 3 |
| 78 | BP | positive regulation of ERK1 and ERK2 cascade | 0.000474806 | 7 |
| 79 | BP | positive regulation of angiogenesis | 0.000489782 | 6 |
| 80 | BP | negative regulation of interferon-gamma production | 0.000518631 | 4 |
| 81 | BP | negative regulation of smooth muscle cell proliferation | 0.000576109 | 4 |
| 82 | BP | apoptotic signaling pathway | 0.000699016 | 5 |
| 83 | BP | regulation of angiogenesis | 0.000702928 | 4 |
| 84 | BP | lipopolysaccharide-mediated signaling pathway | 0.000772478 | 4 |
| 85 | BP | JAK-STAT cascade | 0.000772478 | 4 |
| 86 | BP | growth hormone receptor signaling pathway | 0.000867362 | 3 |
| 87 | BP | positive regulation of isotype switching to IgG isotypes | 0.000867362 | 3 |
| 88 | BP | positive regulation of T-helper 1 type immune response | 0.000867362 | 3 |
| 89 | BP | extrinsic apoptotic signaling pathway in absence of ligand | 0.000924364 | 4 |
| 90 | BP | sexual reproduction | 0.001383727 | 3 |
| 91 | BP | defense response to bacterium | 0.001392016 | 6 |
| 92 | BP | complement activation | 0.001494878 | 5 |
| 93 | BP | negative regulation of interleukin-17 production | 0.001684998 | 3 |
| 94 | BP | positive regulation of tyrosine phosphorylation of Stat1 protein | 0.001684998 | 3 |
| 95 | BP | extrinsic apoptotic signaling pathway | 0.001716328 | 4 |
| 96 | BP | response to estradiol | 0.001764359 | 5 |
| 97 | BP | positive regulation of nitric oxide biosynthetic process | 0.001837539 | 4 |
| 98 | BP | negative regulation of immune response | 0.002014559 | 3 |
| 99 | BP | positive regulation of interleukin-2 biosynthetic process | 0.002014559 | 3 |
| 100 | BP | complement activation, alternative pathway | 0.002372088 | 3 |
| 101 | BP | regulation of interferon-gamma-mediated signaling pathway | 0.002372088 | 3 |
| 102 | BP | positive regulation of nitric-oxide synthase biosynthetic process | 0.002372088 | 3 |
| 103 | BP | positive regulation of transcription, DNA-templated | 0.002541601 | 10 |
| 104 | BP | positive regulation of sequence-specific DNA binding transcription factor activity | 0.002971791 | 5 |
| 105 | BP | positive regulation of T cell differentiation | 0.00316977 | 3 |
| 106 | BP | JAK-STAT cascade involved in growth hormone signaling pathway | 0.00316977 | 3 |
| 107 | BP | cell-cell signaling | 0.003195767 | 7 |
| 108 | BP | positive regulation of chemokine production | 0.004075513 | 3 |
| 109 | BP | cellular response to interferon-gamma | 0.004115234 | 4 |
| 110 | BP | positive regulation of leukocyte chemotaxis | 0.004568129 | 3 |
| 111 | BP | natural killer cell activation | 0.005631312 | 3 |
| 112 | BP | positive regulation of protein phosphorylation | 0.005848028 | 5 |
| 113 | BP | positive regulation of JNK cascade | 0.005946882 | 4 |
| 114 | BP | neutrophil chemotaxis | 0.006204839 | 4 |
| 115 | BP | positive regulation of neutrophil chemotaxis | 0.006796413 | 3 |
| 116 | BP | positive regulation of JAK-STAT cascade | 0.006796413 | 3 |
| 117 | BP | regulation of apoptotic process | 0.00721571 | 6 |
| 118 | BP | cellular response to mechanical stimulus | 0.007594415 | 4 |
| 119 | BP | negative regulation of NF-kappaB transcription factor activity | 0.007594415 | 4 |
| 120 | BP | response to nutrient | 0.008509394 | 4 |
| 121 | BP | nucleotide-binding oligomerization domain containing signaling pathway | 0.008729953 | 3 |
| 122 | BP | positive regulation of interleukin-8 production | 0.009422866 | 3 |
| 123 | BP | positive regulation of vascular endothelial growth factor production | 0.010139499 | 3 |
| 124 | BP | toll-like receptor signaling pathway | 0.010139499 | 3 |
| 125 | BP | negative regulation of interleukin-6 production | 0.010879565 | 3 |
| 126 | BP | negative regulation of T-helper 17 cell differentiation | 0.01128324 | 2 |
| 127 | BP | regulation of natural killer cell proliferation | 0.01128324 | 2 |
| 128 | BP | positive regulation of T cell anergy | 0.01128324 | 2 |
| 129 | BP | polysaccharide assembly with MHC class II protein complex | 0.01128324 | 2 |
| 130 | BP | positive regulation of growth factor dependent skeletal muscle satellite cell proliferation | 0.01128324 | 2 |
| 131 | BP | activation of cysteine-type endopeptidase activity involved in apoptotic process | 0.011630033 | 4 |
| 132 | BP | positive regulation of insulin secretion involved in cellular response to glucose stimulus | 0.011642784 | 3 |
| 133 | BP | antigen processing and presentation of peptide antigen via MHC class I | 0.012428876 | 3 |
| 134 | BP | T cell differentiation | 0.012428876 | 3 |
| 135 | BP | G-protein coupled receptor signaling pathway | 0.012532464 | 12 |
| 136 | BP | movement of cell or subcellular component | 0.012797925 | 4 |
| 137 | BP | positive regulation of I-kappaB kinase/NF-kappaB signaling | 0.013202376 | 5 |
| 138 | BP | positive regulation of epithelial cell migration | 0.01492162 | 3 |
| 139 | BP | protein kinase B signaling | 0.01492162 | 3 |
| 140 | BP | positive regulation of calcidiol 1-monooxygenase activity | 0.016877528 | 2 |
| 141 | BP | antigen processing and presentation of endogenous peptide antigen via MHC class I via ER pathway, TAP-independent | 0.016877528 | 2 |
| 142 | BP | negative regulation of nucleotide-binding oligomerization domain containing 2 signaling pathway | 0.016877528 | 2 |
| 143 | BP | neutrophil apoptotic process | 0.016877528 | 2 |
| 144 | BP | positive regulation of STAT protein import into nucleus | 0.016877528 | 2 |
| 145 | BP | cell maturation | 0.017610363 | 3 |
| 146 | BP | complement activation, classical pathway | 0.018613384 | 4 |
| 147 | BP | negative regulation of tumor necrosis factor production | 0.019508192 | 3 |
| 148 | BP | protein tetramerization | 0.021487854 | 3 |
| 149 | BP | response to ethanol | 0.021715918 | 4 |
| 150 | BP | positive regulation of interferon-alpha biosynthetic process | 0.022440494 | 2 |
| 151 | BP | tyrosine phosphorylation of STAT protein | 0.022440494 | 2 |
| 152 | BP | tyrosine phosphorylation of Stat5 protein | 0.022440494 | 2 |
| 153 | BP | interleukin-2-mediated signaling pathway | 0.022440494 | 2 |
| 154 | BP | regulation of T cell anergy | 0.022440494 | 2 |
| 155 | BP | regulation of vascular endothelial growth factor production | 0.022440494 | 2 |
| 156 | BP | positive regulation of NK T cell proliferation | 0.022440494 | 2 |
| 157 | BP | embryo implantation | 0.023547302 | 3 |
| 158 | BP | monocyte chemotaxis | 0.023547302 | 3 |
| 159 | BP | negative regulation of cell proliferation | 0.024868952 | 7 |
| 160 | BP | positive regulation of apoptotic process | 0.027727772 | 6 |
| 161 | BP | outflow tract morphogenesis | 0.027897537 | 3 |
| 162 | BP | positive regulation of CD8-positive, alpha-beta T cell proliferation | 0.027972311 | 2 |
| 163 | BP | protection from natural killer cell mediated cytotoxicity | 0.027972311 | 2 |
| 164 | BP | cellular response to muramyl dipeptide | 0.027972311 | 2 |
| 165 | BP | positive regulation of toll-like receptor signaling pathway | 0.027972311 | 2 |
| 166 | BP | regulation of defense response to virus by host | 0.027972311 | 2 |
| 167 | BP | positive regulation of natural killer cell activation | 0.027972311 | 2 |
| 168 | BP | organ regeneration | 0.029031858 | 3 |
| 169 | BP | tumor necrosis factor-mediated signaling pathway | 0.029350002 | 4 |
| 170 | BP | response to heat | 0.030184402 | 3 |
| 171 | BP | tyrosine phosphorylation of Stat3 protein | 0.033473153 | 2 |
| 172 | BP | necroptotic signaling pathway | 0.033473153 | 2 |
| 173 | BP | activation-induced cell death of T cells | 0.033473153 | 2 |
| 174 | BP | positive regulation of cAMP metabolic process | 0.033473153 | 2 |
| 175 | BP | leptin-mediated signaling pathway | 0.033473153 | 2 |
| 176 | BP | positive regulation of monocyte chemotactic protein-1 production | 0.038943191 | 2 |
| 177 | BP | negative regulation of B cell activation | 0.038943191 | 2 |
| 178 | BP | positive regulation of interferon-beta biosynthetic process | 0.038943191 | 2 |
| 179 | BP | negative regulation of inflammatory response to antigenic stimulus | 0.038943191 | 2 |
| 180 | BP | regulation of NIK/NF-kappaB signaling | 1.98019802 | 2 |
| 181 | BP | positive regulation of cytosolic calcium ion concentration | 0.040444802 | 4 |
| 182 | BP | cellular response to hydrogen peroxide | 0.041338738 | 3 |
| 183 | BP | protein oligomerization | 0.042660761 | 3 |
| 184 | BP | negative regulation of gene expression | 0.042730319 | 4 |
| 185 | BP | cellular response to organic cyclic compound | 0.043998501 | 3 |
| 186 | BP | response to mechanical stimulus | 0.043998501 | 3 |
| 187 | BP | positive regulation of interleukin-6 biosynthetic process | 0.044382595 | 2 |
| 188 | BP | negative regulation of lipid storage | 0.044382595 | 2 |
| 189 | BP | positive regulation of interleukin-1 beta production | 0.044382595 | 2 |
| 190 | BP | positive regulation of protein kinase C signaling | 0.044382595 | 2 |
| 191 | BP | positive regulation of MHC class II biosynthetic process | 0.044382595 | 2 |
| 192 | BP | positive regulation of smooth muscle cell proliferation | 0.045351743 | 3 |
| 193 | BP | negative regulation of angiogenesis | 0.048103876 | 3 |
| 194 | BP | regulation of inflammatory response | 0.049502344 | 3 |
| 195 | BP | enzyme linked receptor protein signaling pathway | 0.049791535 | 2 |
| 196 | BP | cellular response to leptin stimulus | 0.049791535 | 2 |
| 197 | BP | positive regulation of smooth muscle cell apoptotic process | 0.049791535 | 2 |
| 198 | BP | interleukin-6-mediated signaling pathway | 0.049791535 | 2 |
| 199 | BP | response to molecule of bacterial origin | 0.049791535 | 2 |
| 200 | BP | antigen processing and presentation of exogenous peptide antigen via MHC class I, TAP-independent | 0.049791535 | 2 |
| 201 | CC | external side of plasma membrane | 1.5187E-15 | 18 |
| 202 | CC | extracellular space | 3.77505E-14 | 34 |
| 203 | CC | integral component of lumenal side of endoplasmic reticulum membrane | 2.044E-12 | 9 |
| 204 | CC | extracellular region | 5.42499E-12 | 34 |
| 205 | CC | MHC class II protein complex | 1.6699E-11 | 8 |
| 206 | CC | ER to Golgi transport vesicle membrane | 3.2388E-10 | 9 |
| 207 | CC | transport vesicle membrane | 4.97588E-08 | 7 |
| 208 | CC | clathrin-coated endocytic vesicle membrane | 7.9997E-08 | 7 |
| 209 | CC | integral component of plasma membrane | 1.25569E-06 | 24 |
| 210 | CC | endocytic vesicle membrane | 1.4507E-06 | 7 |
| 211 | CC | trans-Golgi network membrane | 5.59938E-06 | 7 |
| 212 | CC | cell surface | 3.36542E-05 | 13 |
| 213 | CC | lysosomal membrane | 0.000115314 | 9 |
| 214 | CC | plasma membrane | 0.001059757 | 37 |
| 215 | CC | Golgi membrane | 0.001289763 | 11 |
| 216 | CC | MHC class I protein complex | 0.00152534 | 3 |
| 217 | CC | endolysosome membrane | 0.001824003 | 3 |
| 218 | CC | endosome membrane | 0.003238854 | 6 |
| 219 | CC | membrane | 0.005994767 | 22 |
| 220 | CC | interleukin-23 complex | 0.010726424 | 2 |
| 221 | CC | late endosome membrane | 0.017190983 | 4 |
| 222 | CC | transforming growth factor beta receptor homodimeric complex | 0.021338953 | 2 |
| 223 | CC | extracellular exosome | 0.023490645 | 24 |
| 224 | CC | lysosome | 0.033704387 | 5 |
| 225 | CC | integral component of membrane | 0.043487812 | 37 |
| 226 | CC | blood microparticle | 0.048752266 | 4 |
| 227 | MF | cytokine activity | 3.82368E-18 | 19 |
| 228 | MF | peptide antigen binding | 1.9349E-12 | 9 |
| 229 | MF | MHC class II receptor activity | 1.3816E-08 | 6 |
| 230 | MF | growth factor activity | 3.34509E-06 | 9 |
| 231 | MF | chemokine activity | 7.55524E-06 | 6 |
| 232 | MF | transmembrane signaling receptor activity | 2.55834E-05 | 9 |
| 233 | MF | receptor binding | 2.67932E-05 | 11 |
| 234 | MF | heparin binding | 3.09036E-05 | 8 |
| 235 | MF | cytokine receptor binding | 4.5927E-05 | 4 |
| 236 | MF | CXCR3 chemokine receptor binding | 0.000303458 | 3 |
| 237 | MF | interleukin-12 receptor binding | 0.000303458 | 3 |
| 238 | MF | growth hormone receptor binding | 0.000303458 | 3 |
| 239 | MF | interleukin-1 receptor binding | 0.002299217 | 3 |
| 240 | MF | receptor activity | 0.00729564 | 6 |
| 241 | MF | protein homodimerization activity | 0.00736623 | 11 |
| 242 | MF | identical protein binding | 0.008750392 | 11 |
| 243 | MF | interleukin-23 receptor binding | 0.011106102 | 2 |
| 244 | MF | TAP binding | 0.016613302 | 2 |
| 245 | MF | cytokine receptor activity | 0.017092174 | 3 |
| 246 | MF | SMAD binding | 0.02389184 | 3 |
| 247 | MF | MHC class I receptor activity | 0.03295349 | 2 |
| 248 | MF | T cell receptor binding | 0.03295349 | 2 |
| 249 | MF | transforming growth factor beta-activated receptor activity | 0.038340297 | 2 |
| 250 | MF | CCR5 chemokine receptor binding | 0.043697415 | 2 |
| 251 | MF | complement binding | 0.043697415 | 2 |
| 252 | MF | protein heterodimerization activity | 0.045330241 | 7 |
