# Supplementary material for: Integrated Analysis of Key Pathways and Drug Targets Associated With Vogt-Koyanagi-Harada Disease
Source: Front Immunol. 2020 Dec 15;11:587443. doi: 10.3389/fimmu.2020.587443 (PMC7769821; doi:10.3389/fimmu.2020.587443)
Supplement: Supplementary file 1 [file DataSheet_1.zip › Supplementary Table 2.DOCX]

**Supplementary Table S2** The corresponding genes enriched in gene ontology (GO) category shown in Figure 2 were listed in the table.

| Number | GO classification | Pathway | Enriched genes |
| --- | --- | --- | --- |
| 1 | BP | Immune response | CCL8, TGFBR3, HLA-DRB1, IL21, HLA-DRA, KIR2DS2, IL2RA, HLA-DQB1, IFN-γ/IFN Gamma, FAS, HLA-DQA1, CCR6, IL9, HLA-DPB1, FAS3, MCP-1/CCL2, KIR 2DS5, CD40, KIR 3DL1, C3, KIR 3DS1, CXCL9, TNFSF13, IL7, CTLA4, IL6, KIR 2DS1, HLA-B, IL15, HLA-A, HLA-DRB5, HLA-DRB4, ETS1, IL4, CD4, IL37, CXCL1, CXCL13, CXCL10 |
| 2 | BP | Inflammatory response | IL17F, CCL8, NOD1, SPP1, IL23R, IL25, IL2RA, AGER, FAS, IL9, IL23A, FAS3, MCP-1/CCL2, CD40, C3, C3AR1, MIF, TLR3, CXCL9, NLRP1, IL27, C4B, IL6, IL15, IL37, CXCL1, TNFAIP3, CXCL13, TLR9, CXCL10 |
| 3 | BP | Positive regulation of T cell proliferation | IL23R, IL21, IL12B, IL6, IFN-γ/IFN Gamma, IL15, IL23A, HLA-DPB1, IL4, FAS3, CD4, LEP, CD3E |
| 4 | BP | Positive regulation of tyrosine phosphorylation of Stat3 protein | IL6, JAK2, IGHD, IL15, VEGFA, IL23A, STAT3, IL23R, IL21, LEP, IL12B |
| 5 | BP | Interferon-gamma-mediated signaling pathway | JAK1, HLA-DQB1, JAK2, HLA-B, IFN-γ/IFN Gamma, HLA-DQA1, HLA-A, HLA-DRB5, HLA-DRB4, HLA-DPB1, HLA-DRB1, HLA-DRA |
| 6 | BP | Innate immune response | MIF, TLR3, NOD1, NLRP1, IL278, KIR2DS2, C4B, JAK1, AGER, CFI, JAK2, aKIR, HLA-B, KIR 2DS1, IL23A, IRAK1, TLR9, KIR 2DS5, C2, KIR 3DS1 |
| 7 | BP | T cell costimulation | HLA-DQB1, HLA-DQA1, HLA-DRB5, PDCD1/PD1, HLA-DRB4, HLA-DPB1, CD4, HLA-DRB1, HLA-DRA, CTLA4, CD3E |
| 8 | BP | Antigen processing and presentation | HLA-DQB1, HLA-B, IFN-γ/IFN Gamma, HLA-DQA1, HLA-A, HLA-DRB5, HLA-DRB4, HLA-DPB1, HLA-DRB1, HLA-DRA |
| 9 | BP | Positive regulation of interleukin-12 production | AGER, TLR3, IFN-γ/IFN Gamma, IL23A, IL23R, IL12B, TLR9, CD40 |
| 10 | BP | Signal transduction | C38, CCL8, NOD1, STAT3, IL21, KIR2DS2, JAK2, FAS, CCR6, FAS3, MCP-1/CCL2, IRAK1, LEP, KIR 2DS5, KIR 3DL1, C3, KIR 3DS1, TLR3, CXCL9, PDCD1/PD1, TNFSF13, KIR B, KIR 2DS1, IL15, HLA-DRB4, CD4, CXCL1, CXCL10 |
| 11 | CC | External side of plasma membrane | CXCL9, TGFBR2, PDCD1/PD1, TGFBR3, HLA-DRB1, IL12RB2, CTLA4, FAS9, IL6, IFN-γ/IFN Gamma, FAS, HLA-DRB5, HLA-DRB4, IL4, CD4, CD40, CD3E, CXCL10 |
| 12 | CC | Extracellular space | IL17F, CCL8, SPP1, VEGFA, TGFBR3, IL21, IL25, IGHD, IFN-γ/IFN Gamma, IL9, FAS3, MCP-1/CCL2, LEP, CD40, C2, C3, MIF, CXCL9, TNFSF13, IL7, IL12B, CFH, IL278, C4B, CFI, IL6, IL15, HLA-DRB5, IL4, IL37, CXCL1, CXCL13, CFB, CXCL10 |
| 13 | CC | Integral component of lumenal side of endoplasmic reticulum membrane | HLA-DQB1, HLA-B, HLA-DQA1, HLA-A, HLA-DRB5, HLA-DRB4, HLA-DPB1, HLA-DRB1, HLA-DRA |
| 14 | CC | Extracellular region | IL17F, SPP1, VEGFA, TGFBR3, IL25, AGER, IGHD, IFN-γ/IFN Gamma, IL9, IL23A, FAS3, MCP-1/CCL2, LEP, C2, C3, MIF, CXCL9, TNFSF13, IL7, IL12B, CFH, IL278, UACA, C4B, CFI, IL6, IL15, IL4, IL37, CXCL1, CXCL13, TLR9, CFB, CXCL10 |
| 15 | CC | MHC class II protein complex | HLA-DQB1, HLA-DQA1, HLA-A, HLA-DRB5, HLA-DRB4, HLA-DPB1, HLA-DRB1, HLA-DRA |
| 16 | CC | ER to Golgi transport vesicle membrane | HLA-DQB1, HLA-B, HLA-DQA1, HLA-A, HLA-DRB5, HLA-DRB4, HLA-DPB1, HLA-DRB1, HLA-DRA |
| 17 | CC | Transport vesicle membrane | HLA-DQB1, HLA-DQA1, HLA-DRB5, HLA-DRB4, HLA-DPB1, HLA-DRB1, HLA-DRA |
| 18 | CC | Clathrin-coated endocytic vesicle membrane | HLA-DQB1, HLA-DQA1, HLA-DRB5, HLA-DRB4, HLA-DPB1, HLA-DRB1, HLA-DRA |
| 19 | CC | Integral component of plasma membrane | TLR3, TGFBR2, TGFBR3, HLA-DRB1, IL12RB2, HLA-DRA, CTLA4, KIR2DS2, AGER, KIR B, HLA-B, FAS, HLA-DQA1, HLA-A, IL15, CCR6, KIR2DS3, HLA-DRB4, KIR 2DS5, CD40, KIR 3DL1, CD3E, KIR 3DS1, C3AR1 |
| 20 | CC | Endocytic vesicle membrane | HLA-DQB1, HLA-DQA1, HLA-DRB5, HLA-DRB4, HLA-DPB1, HLA-DRB1, HLA-DRA |
| 21 | MF | Cytokine activity | MIF, CXCL9, IL17F, SPP1, VEGFA, TNFSF13, IL7, IL21, IL12B, IL278, IL25, IL6, IFN-γ/IFN Gamma, IL15, IL9, IL23A, IL4, FAS3, IL37 |
| 22 | MF | Peptide antigen binding | HLA-DQB1, HLA-B, HLA-DQA1, HLA-A, HLA-DRB5, HLA-DRB4, HLA-DPB1, HLA-DRB1, HLA-DRA |
| 23 | MF | MHC class II receptor activity | HLA-DQB1, HLA-DQA1, HLA-DRB4, HLA-DPB1, HLA-DRB1, HLA-DRA |
| 24 | MF | Growth factor activity | IL6, IGHD, IL9, VEGFA, IL4, IL7, CXCL1, LEP, IL12B |
| 25 | MF | Chemokine activity | CXCL9, CCL8, CXCL1, MCP-1/CCL2, CXCL13, CXCL10 |
| 26 | MF | Transmembrane signaling receptor activity | KIR2DS2, AGER, TLR3, KIR B, FAS, KIR 2DS1, CD4, TLR9, CD3E |
| 27 | MF | Receptor binding | MIF, JAK1, JAK2, HLA-B, HLA-A, TNFSF13, CXCL1, MCP-1/CCL2, IL278, C3, CXCL10 |
| 28 | MF | Heparin binding | AGER, CCL8, VEGFA, TGFBR3, MCP-1/CCL2, CXCL13, CFH, CXCL10 |
| 29 | MF | Cytokine receptor binding | MIF, IL17F, IL15, IL21 |
| 30 | MF | CXCR3 chemokine receptor binding | JAK2, IL23R, IL12B |
