# Supplementary material for: Integrated Analysis of Key Pathways and Drug Targets Associated With Vogt-Koyanagi-Harada Disease
Source: Front Immunol. 2020 Dec 15;11:587443. doi: 10.3389/fimmu.2020.587443 (PMC7769821; doi:10.3389/fimmu.2020.587443)
Supplement: Supplementary file 1 [file DataSheet_1.zip › Supplementary Table 3.DOCX]

**Supplementary Table S3** All significant pathways of Kyoto Encyclopedia of Genes and Genomes (KEGG) database were listed in the table.

| Number | Pathway of KEGG | P Value | Count |
| --- | --- | --- | --- |
| 1 | Inflammatory bowel disease (IBD) | 2.17522E-21 | 19 |
| 2 | Cytokine-cytokine receptor interaction | 2.08282E-19 | 27 |
| 3 | Allograft rejection | 4.26159E-17 | 14 |
| 4 | Antigen processing and presentation | 7.32093E-17 | 17 |
| 5 | Herpes simplex infection | 2.73245E-16 | 22 |
| 6 | Graft-versus-host disease | 4.59822E-16 | 13 |
| 7 | Type I diabetes mellitus | 1.3484E-14 | 13 |
| 8 | Leishmaniasis | 2.06117E-14 | 15 |
| 9 | Rheumatoid arthritis | 2.17843E-14 | 16 |
| 10 | Autoimmune thyroid disease | 2.29835E-13 | 13 |
| 11 | Staphylococcus aureus infection | 3.75366E-13 | 13 |
| 12 | Intestinal immune network for IgA production | 2.08551E-12 | 12 |
| 13 | Jak-STAT signaling pathway | 3.00611E-12 | 17 |
| 14 | Asthma | 2.02262E-11 | 10 |
| 15 | Epstein-Barr virus infection | 4.69394E-11 | 15 |
| 16 | Influenza A | 5.03042E-11 | 17 |
| 17 | Tuberculosis | 6.52608E-11 | 17 |
| 18 | Toxoplasmosis | 1.70332E-10 | 14 |
| 19 | HTLV-I infection | 1.74161E-09 | 18 |
| 20 | Measles | 1.87435E-09 | 14 |
| 21 | Viral myocarditis | 9.98547E-09 | 10 |
| 22 | Hematopoietic cell lineage | 3.39792E-08 | 11 |
| 23 | Cell adhesion molecules (CAMs) | 4.37528E-08 | 13 |
| 24 | Chagas disease (American trypanosomiasis) | 1.90401E-07 | 11 |
| 25 | Systemic lupus erythematosus | 2.03317E-06 | 11 |
| 26 | Toll-like receptor signaling pathway | 2.3484E-06 | 10 |
| 27 | Pertussis | 1.80162E-05 | 8 |
| 28 | TNF signaling pathway | 2.27823E-05 | 9 |
| 29 | African trypanosomiasis | 2.86334E-05 | 6 |
| 30 | NOD-like receptor signaling pathway | 3.36272E-05 | 7 |
| 31 | Phagosome | 3.99537E-05 | 10 |
| 32 | Malaria | 0.000200084 | 6 |
| 33 | Complement and coagulation cascades | 0.000990912 | 6 |
| 34 | Chemokine signaling pathway | 0.001049653 | 9 |
| 35 | Natural killer cell mediated cytotoxicity | 0.002336381 | 7 |
| 36 | NF-kappa B signaling pathway | 0.002787075 | 6 |
| 37 | Legionellosis | 0.003004699 | 5 |
| 38 | T cell receptor signaling pathway | 0.005074993 | 6 |
| 39 | Hepatitis B | 0.022807137 | 6 |
| 40 | Viral carcinogenesis | 0.026747485 | 7 |
| 41 | Pancreatic cancer | 0.035897469 | 4 |
| 42 | PI3K-Akt signaling pathway | 0.038167045 | 9 |
