# Supplementary material for: Integrated Analysis of Key Pathways and Drug Targets Associated With Vogt-Koyanagi-Harada Disease
Source: Front Immunol. 2020 Dec 15;11:587443. doi: 10.3389/fimmu.2020.587443 (PMC7769821; doi:10.3389/fimmu.2020.587443)
Supplement: Supplementary file 1 [file DataSheet_1.zip › Supplementary Table 4.DOCX]

**Supplementary Table S4** The corresponding genes enriched in Kyoto Encyclopedia of Genes and Genomes (KEGG) pathway shown in Figure 3 were listed in below.

| Number | Pathway of KEGG | Enriched genes |
| --- | --- | --- |
| 1 | Inflammatory bowel disease (IBD) | HLA-DRB4, IL12B, HLA-DRB5, HLA-DPB1, IL12RB2, IL23A, HLA-DQA1, STAT3, HLA-DQB1, IFN-γ/IFN Gamma, IL23R, IL4, IL1B, IL21, IL6, IL17F, HLA-DRB1, HLA-DRA, FOXP3 |
| 2 | Cytokine-cytokine receptor interaction | CXCL10, CXCL1, TNFSF13, MCP-1/CCL2, IL15, CXCL13, IFN-γ/IFN Gamma, IL23R, CCL8, TGFBR2, IL1B, IL21, IL2RA, IL9, CD40, IL12B, IL23A, IL12RB2, IL7, CXCL9, FAS, CCR6, IGHD, LEP, IL4, IL25, IL6 |
| 3 | Allograft rejection | HLA-DRB4, CD40, HLA-DRB5, IL12B, HLA-DPB1, HLA-DQA1, HLA-DQB1, IFN-γ/IFN Gamma, FAS, HLA-B, IL4, HLA-A, HLA-DRB1, HLA-DRA |
| 4 | Antigen processing and presentation | HLA-DRB4, CD4, HLA-DRB5, HLA-DPB1, HLA-DQA1, KIR 2DS5, KIR 3DL1, HLA-DQB1, KIR2DS3, KIR B, KIR 2DS1, KIR2DS2, IFN-γ/IFN Gamma, HLA-B, HLA-A, HLA-DRB1, HLA-DRA |
| 5 | Herpes simplex infection | HLA-DRB4, IL12B, HLA-DRB5, HLA-DPB1, HLA-DQA1, HLA-DQB1, TLR9, MCP-1/CCL2, JAK1, IL15, JAK2, TRAF5, FAS, IFN-γ/IFN Gamma, C3, C3, HLA-B, IL1B, IL6, HLA-A, HLA-DRB1, HLA-DRA |
| 6 | Graft-versus-host disease | HLA-DRB4, HLA-DRB5, FAS, IFN-γ/IFN Gamma, HLA-DPB1, HLA-DQA1, HLA-DQB1, HLA-B, IL1B, IL6, HLA-A, HLA-DRB1, HLA-DRA |
| 7 | Type I diabetes mellitus | HLA-DRB4, HLA-DRB5, IL12B, FAS, IFN-γ/IFN Gamma, HLA-DPB1, HLA-DQA1, HLA-DQB1, HLA-B, IL1B, HLA-A, HLA-DRB1, HLA-DRA |
| 8 | Leishmaniasis | HLA-DRB4, IL12B, HLA-DRB5, HLA-DPB1, HLA-DQA1, HLA-DQB1, JAK1, JAK2, IFN-γ/IFN Gamma, C3, IL4, IL1B, IRAK1, HLA-DRB1, HLA-DRA |
| 9 | Rheumatoid arthritis | HLA-DRB4, HLA-DRB5, HLA-DPB1, IL23A, HLA-DQA1, HLA-DQB1, TNFSF13, MCP-1/CCL2, IL15, IFN-γ/IFN Gamma, VEGFA, IL1B, IL6, HLA-DRB1, CTLA4, HLA-DRA |
| 10 | Autoimmune thyroid disease | HLA-DRB4, HLA-DRB5, CD40, FAS, HLA-DPB1, HLA-DQA1, HLA-DQB1, IL4, HLA-B, HLA-A, HLA-DRB1, HLA-DRA, CTLA4 |
