# Supplementary material for: Integrated Analysis of Key Pathways and Drug Targets Associated With Vogt-Koyanagi-Harada Disease
Source: Front Immunol. 2020 Dec 15;11:587443. doi: 10.3389/fimmu.2020.587443 (PMC7769821; doi:10.3389/fimmu.2020.587443)
Supplement: Supplementary file 1 [file DataSheet_1.zip › Supplementary Table 5.DOCX]

**Supplementary Table S5** The descriptions of genes symbol and details information shown in Figure 7 were listed as follow.

| Number | Gene Symbol | Description | MNC | Degree | Closeness | Radiality | EcCentricity | EPC |
| --- | --- | --- | --- | --- | --- | --- | --- | --- |
| 1 | IL6 | Interleukin-6 | 57 | 58 | 71.5 | 4.7093 | 0.33333 | 37.751 |
| 2 | CTLA4 | cytotoxic T-lymphocyte associated protein 4 | 45 | 45 | 64.66667 | 4.53488 | 0.33333 | 36.022 |
| 3 | IL4 | Interleukin-4 | 45 | 45 | 64.5 | 4.52326 | 0.33333 | 36.561 |
| 4 | IFNG | interferon gamma | 45 | 45 | 64.16667 | 4.5 | 0.33333 | 36.187 |
| 5 | IL1B | Interleukin-1 beta | 43 | 43 | 63.5 | 4.5 | 0.33333 | 35.954 |
| 6 | STAT3 | signal transducer and activator of transcription 3 | 41 | 42 | 63 | 4.48837 | 0.33333 | 34.841 |
| 7 | CCL2 | C-C motif chemokine ligand 2 | 40 | 40 | 61.66667 | 4.44186 | 0.33333 | 35.698 |
| 8 | CD40 | CD40 molecule | 40 | 40 | 61.83333 | 4.45349 | 0.33333 | 35.607 |
| 9 | FOXP3 | Forkhead box protein P3 | 38 | 38 | 61.33333 | 4.46512 | 0.33333 | 34.727 |
| 10 | IL2RA | Interleukin-2 receptor subunit alpha | 38 | 38 | 60.66667 | 4.4186 | 0.33333 | 34.562 |
| 11 | IL15 | Interleukin-15 | 36 | 36 | 59.5 | 4.38372 | 0.33333 | 33.76 |
| 12 | CXCL10 | C-X-C motif chemokine 10 | 35 | 35 | 59.16667 | 4.38372 | 0.33333 | 35.094 |
| 13 | TLR3 | Toll-like receptor 3 | 35 | 35 | 59.33333 | 4.39535 | 0.33333 | 33.569 |
| 14 | VEGFA | Vascular endothelial growth factor A | 34 | 34 | 59 | 4.39535 | 0.33333 | 33.645 |
| 15 | TLR9 | toll like receptor 9 | 33 | 33 | 58.33333 | 4.37209 | 0.33333 | 33.757 |
| 16 | IL7 | Interleukin-7 | 32 | 32 | 57.5 | 4.33721 | 0.33333 | 33.612 |
| 17 | CXCL1 | Growth-regulated alpha protein | 31 | 31 | 56.66667 | 4.30233 | 0.33333 | 32.762 |
| 18 | GPR29 | C-C motif chemokine receptor 6 | 29 | 29 | 55.83333 | 4.2907 | 0.33333 | 31.965 |
| 19 | IL23R | interleukin 23 receptor | 27 | 28 | 55.83333 | 4.31395 | 0.33333 | 30.218 |
| 20 | JAK2 | Janus kinase 2 | 27 | 27 | 54.83333 | 4.26744 | 0.33333 | 31.546 |
| 21 | CXCL9 | C-X-C motif chemokine 9 | 27 | 27 | 55 | 4.27907 | 0.33333 | 31.656 |
| 22 | IL9 | Interleukin-9 | 26 | 26 | 54.16667 | 4.24419 | 0.33333 | 32.857 |
| 23 | PDCD1 | programmed cell death 1 | 26 | 26 | 54.41667 | 4.25581 | 0.25 | 29.595 |
| 24 | PTPN22 | protein tyrosine phosphatase non-receptor type 22 | 24 | 25 | 53.66667 | 4.23256 | 0.33333 | 25.867 |
| 25 | IL21 | Interleukin-21 | 24 | 25 | 53.83333 | 4.24419 | 0.33333 | 30.264 |
| 26 | JAK1 | Janus kinase 1 | 24 | 24 | 53.33333 | 4.23256 | 0.33333 | 30.8 |
| 27 | HLA-DRB1 | HLA class II histocompatibility antigen, DRB1-14 beta chain | 23 | 23 | 52.75 | 4.2093 | 0.25 | 27.543 |
| 28 | IL17F | interleukin 17F | 23 | 23 | 52.5 | 4.19767 | 0.33333 | 29.991 |
| 29 | IL23A | Interleukin-23 subunit alpha | 22 | 22 | 51.83333 | 4.17442 | 0.33333 | 28.854 |
| 30 | CD4 | T-cell surface glycoprotein CD4 | 21 | 22 | 52.08333 | 4.18605 | 0.25 | 28.639 |
| 31 | CXCL13 | C-X-C motif chemokine 13 | 22 | 22 | 52.66667 | 4.23256 | 0.33333 | 29.652 |
| 32 | HLA-A | MHC class I antigen | 21 | 21 | 51.58333 | 4.17442 | 0.25 | 24.337 |
| 33 | C3 | complement C3 | 19 | 20 | 50.16667 | 4.10465 | 0.33333 | 23.939 |
| 34 | LEP | Leptin | 20 | 20 | 50.91667 | 4.15116 | 0.25 | 27.299 |
| 35 | IL12RB2 | interleukin 12 receptor subunit beta 2 | 20 | 20 | 50.75 | 4.13953 | 0.25 | 28.042 |
| 36 | CD3E | T-cell surface glycoprotein CD3 epsilon chain | 20 | 20 | 51.25 | 4.17442 | 0.25 | 25.048 |
| 37 | TNFAIP3 | TNF alpha induced protein 3 | 19 | 19 | 51.16667 | 4.19767 | 0.33333 | 25.509 |
| 38 | FAS | Tumor necrosis factor receptor superfamily member 6 | 18 | 18 | 50.08333 | 4.13953 | 0.25 | 27.52 |
| 39 | HLA-DQA1 | HLA class II histocompatibility antigen, DQ alpha 1 chain | 16 | 17 | 49.41667 | 4.11628 | 0.25 | 22.012 |
| 40 | HLA-DQB1 | HLA class II histocompatibility antigen, DQ beta 1 chain | 16 | 16 | 48 | 4.03488 | 0.25 | 21.779 |
| 41 | HLA-B | HLA class I histocompatibility antigen B alpha chain | 16 | 16 | 48.16667 | 4.04651 | 0.25 | 17.414 |
| 42 | IRAK1 | Interleukin-1 receptor-associated kinase 1 | 16 | 16 | 48.58333 | 4.06977 | 0.25 | 24.256 |
| 43 | HLA-DRA | major histocompatibility complex, class II, DR alpha | 15 | 15 | 47.16667 | 3.98837 | 0.25 | 21.08 |
| 44 | CCL8 | C-C motif chemokine 8 | 14 | 14 | 46.66667 | 3.98837 | 0.25 | 23.55 |
| 45 | IL12B | interleukin 12B | 13 | 13 | 46.83333 | 4.02326 | 0.25 | 21.098 |
| 46 | C3AR1 | C3a anaphylatoxin chemotactic receptor | 13 | 13 | 45.5 | 3.93023 | 0.25 | 17.862 |
| 47 | SPP1 | Osteopontin | 12 | 12 | 46.08333 | 4 | 0.25 | 20.637 |
| 48 | CFB | complement factor B | 11 | 11 | 45.33333 | 3.96512 | 0.25 | 14.595 |
| 49 | HLA-DRB5 | major histocompatibility complex, class II, DR beta 5 | 11 | 11 | 41.66667 | 3.68605 | 0.25 | 15.42 |
| 50 | HLA-DPB1 | HLA class II histocompatibility antigen, DRB1-1 beta chain | 11 | 11 | 41.66667 | 3.68605 | 0.25 | 15.738 |
| 51 | MIF | Macrophage migration inhibitory factor | 11 | 11 | 45.16667 | 3.95349 | 0.25 | 19.763 |
| 52 | CFH | complement factor H | 10 | 10 | 44.58333 | 3.94186 | 0.25 | 14.087 |
| 53 | C4B | Complement C4-B | 10 | 10 | 45.16667 | 3.97674 | 0.25 | 11.918 |
| 54 | IL27 | interleukin 27 | 10 | 10 | 44.75 | 3.95349 | 0.25 | 18.251 |
| 55 | IL37 | Interleukin-37 | 9 | 9 | 43.91667 | 3.90698 | 0.25 | 17.835 |
| 56 | ETS1 | ETS proto-oncogene 1, transcription factor | 8 | 9 | 44.91667 | 3.98837 | 0.25 | 16.43 |
| 57 | NOD1 | Nucleotide-binding oligomerization domain-containing protein 1 | 9 | 9 | 42.75 | 3.82558 | 0.25 | 16.325 |
| 58 | CFI | complement factor I | 7 | 7 | 41.66667 | 3.7907 | 0.25 | 9.618 |
| 59 | TRAF5 | TNF receptor associated factor 5 | 6 | 7 | 42.25 | 3.83721 | 0.25 | 12.604 |
| 60 | GH1 | growth hormone 1 | 7 | 7 | 41.25 | 3.76744 | 0.25 | 14.141 |
| 61 | IL25 | Interleukin-25 | 7 | 7 | 42.16667 | 3.83721 | 0.25 | 15.653 |
| 62 | AGER | Advanced glycosylation end product-specific receptor | 7 | 7 | 42.33333 | 3.84884 | 0.25 | 15.925 |
| 63 | C2 | complement C2 | 6 | 6 | 35.33333 | 3.34884 | 0.25 | 7.133 |
| 64 | TGFBR2 | TGF-beta receptor type-2 | 5 | 6 | 42.33333 | 3.87209 | 0.25 | 10.435 |
| 65 | FCRL3 | Fc receptor like 3 | 5 | 6 | 41.08333 | 3.77907 | 0.25 | 11.865 |
| 66 | NLRP1 | NLR family pyrin domain containing 1 | 6 | 6 | 42.58333 | 3.88372 | 0.25 | 12.238 |
| 67 | BCL2A1 | Bcl-2-related protein A1 | 6 | 6 | 41.58333 | 3.81395 | 0.25 | 11.529 |
| 68 | EGR2 | early growth response 2 | 3 | 5 | 39.25 | 3.67442 | 0.25 | 7.115 |
| 69 | CLEC16A | C-type lectin domain containing 16A | 5 | 5 | 40 | 3.72093 | 0.25 | 9.221 |
| 70 | BACH2 | BTB domain and CNC homolog 2 | 5 | 5 | 40.08333 | 3.72093 | 0.25 | 8.586 |
| 71 | KIR3DL1 | killer cell immunoglobulin like receptor, three Ig domains and long cytoplasmic tail 1 | 4 | 4 | 39.33333 | 3.69767 | 0.25 | 8.039 |
| 72 | KIR2DL4 | killer cell immunoglobulin like receptor, two Ig domains and long cytoplasmic tail 4 | 4 | 4 | 39.33333 | 3.69767 | 0.25 | 7.902 |
| 73 | FGFR1OP | centrosomal protein 43 | 2 | 4 | 39.41667 | 3.69767 | 0.25 | 8.631 |
| 74 | TYR | Tyrosinase | 2 | 4 | 40 | 3.75581 | 0.25 | 6.565 |
| 75 | DAB2 | Disabled homolog 2 | 2 | 3 | 37.08333 | 3.55814 | 0.25 | 5.635 |
| 76 | ADO | 2-aminoethanethiol dioxygenase | 2 | 3 | 32.66667 | 3.18605 | 0.25 | 2.762 |
| 77 | PAX3 | Paired box protein Pax-3 | 1 | 3 | 31.95 | 3.17442 | 0.2 | 3.056 |
| 78 | C1orf141 | chromosome 1 open reading frame 141 | 2 | 3 | 34.83333 | 3.39535 | 0.25 | 3.271 |
| 79 | ZNF365 | zinc finger protein 365 | 2 | 2 | 26.66667 | 2.60465 | 0.2 | 1.362 |
| 80 | TGFBR3 | transforming growth factor beta receptor 3 | 1 | 1 | 28.31667 | 2.88372 | 0.2 | 1.624 |
| 81 | HOXB3 | homeobox B3 | 1 | 1 | 26.85 | 2.68605 | 0.2 | 1.462 |
| 82 | TNFSF13 | Tumor necrosis factor ligand superfamily member 13 | 1 | 1 | 28.18333 | 2.84884 | 0.2 | 1.895 |
| 83 | SUMO4 | small ubiquitin like modifier 4 | 1 | 1 | 32.41667 | 3.24419 | 0.25 | 2.81 |
| 84 | AKIRIN2 | akirin 2 | 1 | 1 | 38.58333 | 3.72093 | 0.25 | 3.724 |
| 85 | CYP2R1 | cytochrome P450 family 2 subfamily R member 1 | 1 | 1 | 27.31667 | 2.76744 | 0.2 | 1.553 |
| 86 | EBI3 | Epstein-Barr virus induced 3 | 1 | 1 | 35.66667 | 3.5 | 0.25 | 3.185 |
| 87 | KIAA1109 | KIAA1109 | 1 | 1 | 32.5 | 3.25581 | 0.25 | 2.905 |
