# Supplementary material for: Integrated Analysis of Key Pathways and Drug Targets Associated With Vogt-Koyanagi-Harada Disease
Source: Front Immunol. 2020 Dec 15;11:587443. doi: 10.3389/fimmu.2020.587443 (PMC7769821; doi:10.3389/fimmu.2020.587443)
Supplement: Supplementary file 1 [file DataSheet_1.zip › Supplementary Table 6.DOCX]

**Supplementary Table S6** All drugs identified by the DGidb analysis.

| Number | Target Gene | DGidb Drug | Score | Sources | PMID | Interaction Type |
| --- | --- | --- | --- | --- | --- | --- |
| 1 | IFNG | FUMARIC ACID | 1 | NCI | None found | None found |
| 2 | IFNG | APREMILAST | 1 | Drugbank | None found | immunosuppressive agents |
| 3 | IFNG | DOXIFLURIDINE | 2 | NCI | 12553027 | None found |
| 4 | IFNG | TRETINOIN | 2 | NCI | 9792441 | None found |
| 5 | IFNG | AMIKACIN | 2 | NCI | 8913387 | None found |
| 6 | IFNG | AMITRIPTYLINE | 2 | NCI | 10996474 | None found |
| 7 | IFNG | CYCLOPHOSPHAMIDE | 2 | NCI | 10823419 | drug/small molecule |
| 8 | IFNG | GANCICLOVIR | 2 | NCI | 2992368 | antiviral agents |
| 9 | IFNG | IBUPROFEN | 2 | NCI | 8931897 | cyclooxygenase inhibitors |
| 10 | IFNG | MELATONIN | 2 | NCI | 7722866 | tryptamines |
| 11 | IFNG | MELPHALAN | 2 | NCI | 9864423 | immunosuppressive agents |
| 12 | IFNG | METHYLPREDNISOLONE | 2 | NCI | 9396353 | glucocorticoids |
| 13 | IFNG | PEFLOXACIN | 2 | NCI | 8673851 | topoisomerase inhibitors |
| 14 | IFNG | PREDNISONE | 2 | NCI | 10784002 | immunosuppressive agents |
| 15 | IFNG | INTERLEUKIN 1BETA | 2 | NCI | 8383325 | None found |
| 16 | IFNG | SURAMIN | 2 | NCI | 1899122 | antiparasitic agents/ antineoplastic agents |
| 17 | IFNG | THEOPHYLLINE | 2 | NCI | 11918854 | phosphodiesterase inhibitors |
| 18 | IFNG | THROMBIN | 2 | NCI | 9731747 | None found |
| 19 | IFNG | TRASTUZUMAB | 2 | NCI | 15297404 | antineoplastic and immunomodulating agents |
| 20 | IFNG | URSODIOL | 2 | NCI | 10784002 | cytochrome p-450 cyp2e1 inhibitors |
| 21 | IFNG | BLEOMYCIN (CHEMBL3039590) | 2 | NCI | 8556994 | antineoplastic agents |
| 22 | IFNG | OLSALAZINE | 3 | Drugbank | 17034586/ 9797390 | sensory system agents |
| 23 | IFNG | FONTOLIZUMAB | 4 | TdgClinicalTrial/ Chembllnteractions/ Drugbank/ TTD | None found | Antibody/ inhibitor |
| 24 | IFNG | GLUCOSAMINE | 5 | Drugbank | 16155294/ 16431966/ 16709188/ 17238806 | None found |
| 25 | IFNG, IL6 | INTERFERON ALFA-2B | 3 | NCI | 10540707/ 9192993 | immunomodulatory agents |
| 26 | IL6 | VX-702 | 1 | Drugbank | None found | antiinflammatory agent/ cardiovascular agent |
| 27 | IL6 | CDP-6038 | 1 | TdgClinicalTrial | None found | None found |
| 28 | IL6 | SIRUKUMAB | 1 | Chembllnteractions | None found | inhibitor |
| 29 | IL6 | OLOKIZUMAB | 1 | Chembllnteractions | None found | inhibitor |
| 30 | IL6 | ELSILIMOMAB | 1 | Chembllnteractions | None found | inhibitor |
| 31 | IL6 | PF-04236921 | 1 | Chembllnteractions | None found | inhibitor |
| 32 | IL6 | IBUDILAST | 2 | TTD/ Drugbank | None found | inhibitor |
| 33 | IL6 | ARSENIC TRIOXIDE | 2 | NCI | 16638192 | antineoplastic and immunomodulating agents |
| 34 | IL6 | MIDOSTAURIN | 2 | NCI | 10463595 | Kinase Inhibitors |
| 35 | IL6 | DIHYDROSPINGOSINE | 2 | NCI | 10022508 | None found |
| 36 | IL6 | FENTANYL | 2 | NCI | 9527747 | serotonin modulators |
| 37 | IL6 | GALLIUM NITRATE | 2 | NCI | 8788232 | immunosuppressive agents |
| 38 | IL6 | GEMFIBROZIL | 2 | NCI | 8941582 | lipid regulating agents |
| 39 | IL6 | IFOSFAMIDE | 2 | NCI | 9260581 | immunosuppressive agents |
| 40 | IL6 | LEVOFLOXACIN | 2 | NCI | 12714806 | topoisomerase inhibitors |
| 41 | IL6 | LINEZOLID | 2 | NCI | 14561977 | None found |
| 42 | IL6 | METRONIDAZOLE | 2 | NCI | 12111578 | cytochrome p-450 cyp3a inhibitors |
| 43 | IL6 | NELFINAVIR | 2 | NCI | 15388451 | None found |
| 44 | IL6 | SAQUINAVIR | 2 | NCI | 15388451 | j05ae(protease inhibitors) |
| 45 | IL6 | VITAMIN K | 2 | NCI | 12053098 | None found |
| 46 | IL6 | CLAZAKIZUMAB | 2 | TdgClinicalTrial/ Chembllnteractions | None found | inhibitor |
| 47 | IL6 | GINSENG, ASIAN | 3 | Drugbank | 17436372/ 14642426 | antagonist |
| 48 | IL6 | SILTUXIMAB | 4 | MyCancerGenome/ Chembllnteractions/ Drugbank | None found | antagonist/ antibody/ inhibitor |
